# Dissecting the RAD51–BRC4 Interaction Landscape through Integrative Molecular Simulations and Experimental Biophysics

**DOI:** 10.1021/acs.jcim.5c01639

**Published:** 2025-10-22

**Authors:** Veronica Bresciani, Francesco Rinaldi, Pedro Franco, Stefania Girotto, Andrea Cavalli, Julian D. Langer, Matteo Masetti, Mattia Bernetti

**Affiliations:** † Computational and Chemical Biology, 121451 Istituto Italiano di Tecnologia, Genova 16163, Italy; ‡ Department of Pharmacy and Biotechnology (FABIT), 9296 Alma Mater Studiorum— Università di Bologna, Bologna 40126, Italy; § 28273 Max Planck Institute of Biophysics, Max-von-Laue-Strasse 3, Frankfurt am Main 60438, Germany; ∥ Max Planck Institute for Brain Research, Max-von-Laue-Strasse 4, Frankfurt am Main 60438, Germany; ⊥ Centre Européen de Calcul Atomique et Moléculaire (CECAM), Ecole Polytechnique Fédérale de Lausanne, Lausanne 1015, Switzerland; # Structural Biophysics Facility, Istituto Italiano di Tecnologia, Genoa 16163, Italy; ¶ Department of Biomolecular Sciences (DISB), 19044 Università degli Studi di Urbino “Carlo Bo”, Urbino 61029, Italy

## Abstract

The interaction between the RAD51 and BRCA2 proteins is central to homologous recombination (HR), a crucial pathway ensuring high-fidelity DNA repair. Recruitment of RAD51 involves eight highly conserved regions on BRCA2, named BRC repeats. The interaction between the fourth BRC repeat (BRC4) and the RAD51 *C*-terminal domain has been structurally characterized, while the complex of full-length RAD51 with the peptide remains elusive. This gap limits our understanding of cytosolic RAD51 recruitment driven by the BRCA2 BRC repeats, which is one of the first crucial steps in HR. Here, we report an integrative experimental and in silico approach to reconstruct the conformational ensemble in solution for full-length RAD51 in complex with BRC4. We combined AlphaFold2, cross-linking mass spectrometry, and small-angle X-ray scattering data with molecular dynamics simulations. Our results show that the full-length RAD51–BRC4 complex is a mixture of compact and elongated conformations and allow for the identification of key residues at the interface between the RAD51 *N*-terminus and BRC4, mediating complex conformational dynamics. Our evidence provides robust atomic-level insights into the RAD51–BRC4 interaction, shedding light on the molecular features that govern the recognition between these two proteins, while unveiling novel hotspots for developing novel anticancer agents.

## Graphical abstract

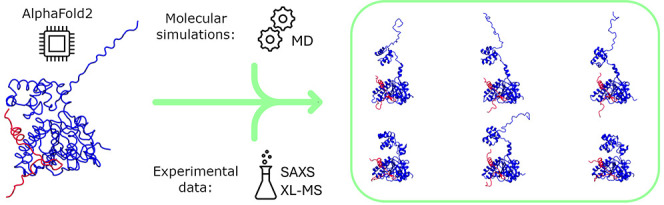

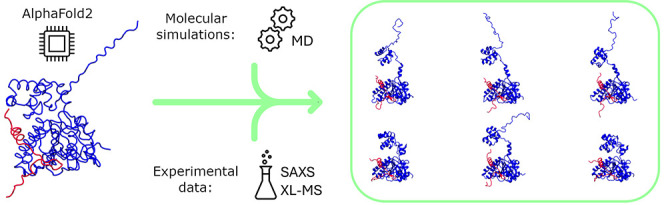

## Introduction

Homologous recombination (HR) is an essential process of synthesis/growth 2 (S/G2) phases, ensuring the high fidelity fixing of very deleterious DNA lesions known as double-strand breaks.^1^ HR requires a series of well-coordinated steps involving multiple proteins.^2^ Among these, BRCA2 and RAD51 play a crucial role.^3^ RAD51 is a recombinase that displays the tendency to assemble into fibril-like oligomers, which are fundamental for enabling strand exchange, an essential step of HR.^4–6^ Although the three-dimensional structure of BRCA2 has not been solved yet, biochemical evidence has proved that it can interact with RAD51 through eight highly conserved sequences, referred to as BRC repeats.^3,7,8^ Very limited structural information is available on this complex. However, BRC repeats are believed to act synergistically, allowing for the recruitment of RAD51 in the cytosol and directing it to damaged DNA sites.^7,9^ X-ray crystallography studies have elucidated the interaction between the RAD51 *C*-terminal (*C*-ter) domain and the fourth BRC repeat (BRC4), which was reported to exhibit the highest affinity for RAD51 (PDB-ID: 1N0W).^9^ While this work identified two domains, namely, the FXXA and LFDE, as crucial for RAD51–BRC4 binding,^9,10^ the absence of the RAD51 *N*-terminal domain (*N*-ter) in the solved structure limits our knowledge of the structural changes induced by BRC repeats binding in this region.^3,11,12^ Recently, we suggested that BRC4 binding triggers a rearrangement of the RAD51 *N*-terminal into an intrinsically disordered region, which might explain the difficulties encountered in obtaining the X-ray crystal structure of the full RAD51–BRC4 complex.^11^ Characterizing these partially structured states is fundamental, as it represents a key step toward understanding the dynamic interplay between RAD51’s plasticity and its functional role in DNA repair. However, achieving high-resolution structural insights into biomolecules that exhibit highly flexible behavior, such as intrinsically disordered proteins, is particularly challenging,^11^ since these systems are better described as structural ensembles rather than single conformations.^11^ In this context, the integration of low-resolution experimental techniques with molecular simulations provides an effective strategy to overcome the limitations of each method, enabling the accurate reconstruction of atomic-scale conformational ensembles in solution.^13–17^

In this work, we integrate state-of-the-art computational approaches with experimental data from small-angle X-ray scattering (SAXS) and cross-linking mass spectrometry (XL-MS) to reconstruct, for the first time, the conformational ensemble of the RAD51–BRC4 complex in solution (Figure 1). Our protocol leverages artificial intelligence methods, namely, AlphaFold2, to generate an initial guess of the RAD51–BRC4 complex. The conformational dynamics of this model is then explored via enhanced-sampling molecular dynamics (MD) approaches, and the structural ensemble of the RAD51–BRC4 complex is finally reconstructed by integrating SAXS and XL-MS experimental data in this pipeline (Figure 1). Crucially, we demonstrate that accounting for solvent contribution is essential for reconciling MD-derived structures with SAXS spectra, enabling a single-structure fit. We then integrate information from XL-MS data into enhanced sampling simulations to effectively explore the system’s conformational landscape. The resulting ensemble, reweighted according to the maximum entropy (maxent) principle to improve consistency with SAXS data, reveals a dynamic equilibrium between compact and elongated conformations, shedding light on the structural plasticity of the RAD51–BRC4 complex. Notably, this comprehensive characterization uncovers specific residues at the interface of BRC4 and RAD51’s *N*-ter domain that play key roles in stabilizing their binding, providing new insights with potential applications in targeting this interaction within synthetically lethal therapeutic contexts.^18^

**Figure 1:**
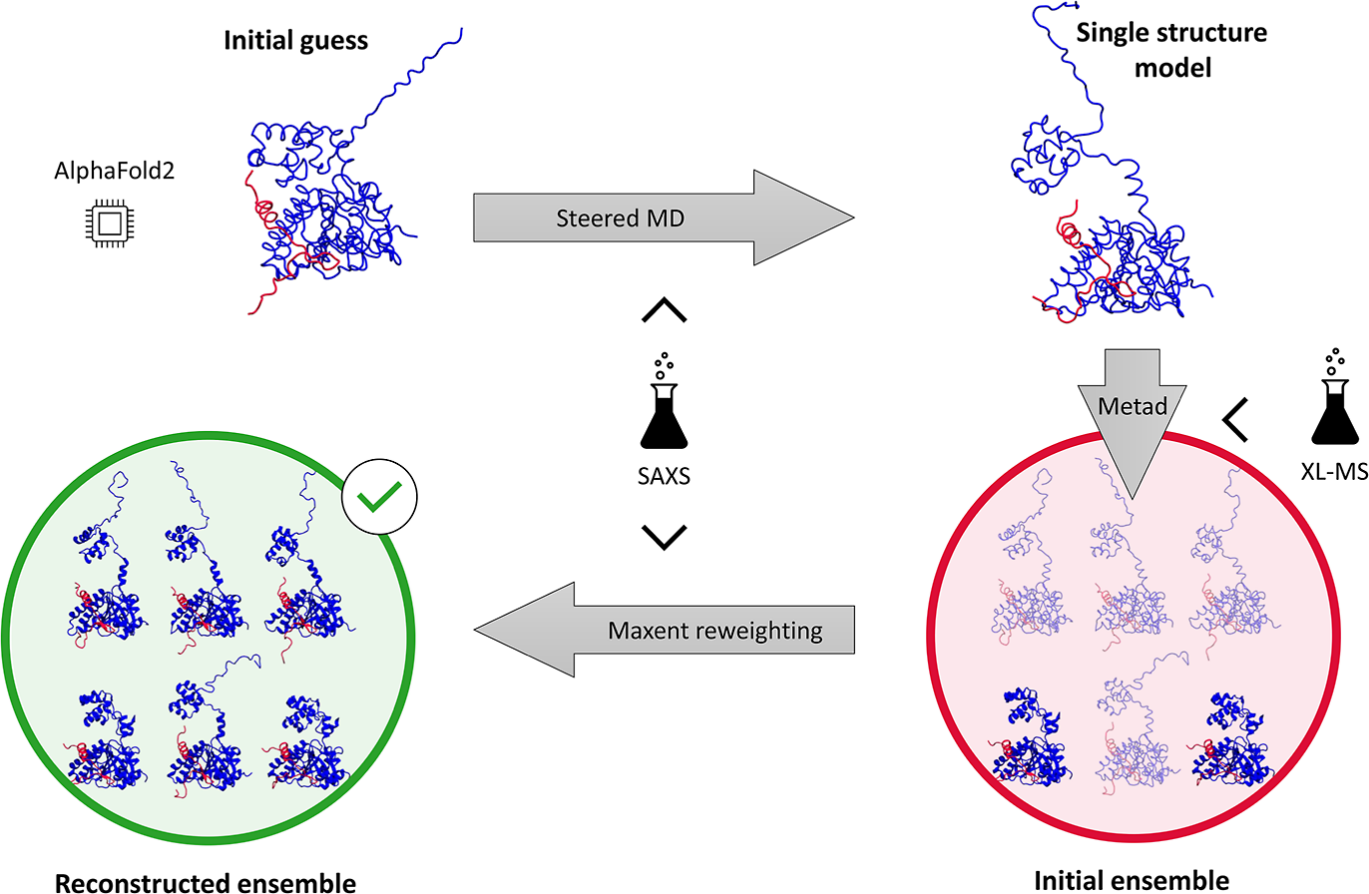
Schematic depiction of the pipeline presented in this work. An initial structural guess for the full RAD51–BRC4 complex is generated through the structure prediction tool AlphaFold2. Steered MD simulations (upper horizontal arrow) are then used to increase the compatibility of this single structure with reference SAXS experimental data. Metadynamics simulations (vertical arrow) started from the improved single-structure model are used to generate an ensemble of diverse configurations of the RAD51–BRC4 complex (red circle, different shading for the structures denotes different weights). Newly generated XL-MS data are integrated at this stage to optimize sampling. Finally, the generated ensemble (prior, red circle) is reweighted through the maximum entropy principle (lower horizontal arrow) to identify an ensemble of structures (posterior, green circle) in agreement with experimental SAXS data.

## Methods

### His-RAD51 [F86E, A89E] Expression, Purification, and Peptide Synthesis

Monomeric His-RAD51 [F86E, A89E] was expressed and purified as previously described in ref 11. BRC4 peptide (NH_2_-KEPTLLGFHTASGKKVKIAKESLDKVKNLFDEKEQ-COOH) was synthesized by Thermofisher Scientific, while biotinylated BRC4 (BioBRC4) (Bio-Ahx-KEPTLLGFHTASGKKVKIAKESLDKVKNLFDEKEQ-COOH) was synthesized by Peptide Protein Research Ltd.

### Static Light Scattering

Static light scattering (SLS) analyses were performed on a Viscotek GPCmax/TDA (Malvern, UK) instrument, connected in tandem with a series of two TSKgel G3000PWxl size-exclusion chromatography columns (Tosoh Bioscience) as already described in ref 11. For all the experiments, the system was equilibrated with buffer containing 20 mM Hepes pH 8.00, 100 mM Na_2_SO_4_, and 5% glycerol. Monomeric RAD51 [F86E, A89E] at 24 μM (0.94 mg/mL) was incubated for 1 h on a thermo-block at 25 °C, in the absence (buffer only) or presence of BRC4 or biotinylated BRC4 peptides (respectively dissolved in the running buffer (stock concentration 1 mM) and in 100% DMSO (stock concentration 2 mM)) in a 4-fold higher stoichiometric excess (final DMSO concentration for BioBRC4 = 5%). Data analysis was performed using Viscotek software, calibrating the instrument with Bovine Serum Albumin at 5 mg/mL. Data were exported as .csv files and regraphed using GraphPad Prism 10.

### Biolayer Interferometry

Biolayer interferometry (BLI) experiments were performed by utilizing an Octet K2 system (Sartorius). All BLI experiments were carried out in an assay buffer containing 20 mM Hepes, pH 8.00, 100 mM Na_2_SO_4_, 5% glycerol, 0.05% Tween 20, 0.1% PEG8000, and 0.5 mM Sodium Deoxycholate. The following protocol was applied: 60 s baseline, 240 s loading, 240 s baseline, 180 s association, 180 s dissociation. For every step, shaking at 1000 rpm was enabled, and the temperature was set at 25 °C. Streptavidin Octet biosensors (18-5019, Sartorius) were initially dipped into assay buffer to record an initial baseline for 60 s. BioBRC4 was solubilized in 100% DMSO at a 2 mM concentration, diluted at a final 1 μM concentration in assay buffer, and then immobilized to the Streptavidin sensor through a 240 s loading step. After the loading stage, a second baseline of 240 s was recorded in wells containing only assay buffer to verify the stability of the signal and remove unbound peptide. For each experiment, sensors were subsequently dipped for 180 s into wells containing His-RAD51 [F86E, A89E] (460, 230, and 115 nM) to measure association signals and finally moved to wells containing only assay buffer to assess complex dissociation. Two replicates were run for each His-RAD51 [F86E, A89E] concentration. BLI experiments were carried out, including a double reference: a reference well (where only immobilized bioBRC4 was present on the sensor and no analyte (0 nM) during association) and a reference sensor (where no bioBRC4 was immobilized on the sensor and His-RAD51 [F86E, A89E] concentration was matched during association). Data were analyzed using Octet Analysis Studio 12.2 by subtracting, from recorded sensorgrams, the signals of both the reference well and reference sensors to remove the signals due to nonspecific binding. The recorded sensorgrams were corrected by aligning to the average of the second baseline steps and applying Savitzky–Golay filtering and interstep correction based on the second baseline step. To calculate *R*_max_, the response at the end of the association phase (170–175 s) was extrapolated. All data presented were exported to .csv files and regraphed using GraphPad Prism 10.

### Cross-Linking: Sample Preparation and Reaction Conditions Setup

For cross-linking with bis­(sulfosuccinimidyl)­suberate (BS3), the purified His-RAD51­[F86E, A89E] concentration was adjusted to 24 μM (=0.98 mg/mL). It was mixed with an equimolar concentration of biotinylated BRC4 peptide (previously solubilized as a 2 mM stock in 100% DMSO), with a final DMSO concentration lower than 5% (v/v). The mixture was incubated for 30 min at 20 °C on a thermoblock to allow for complex formation and then cross-linked with a final concentration of 1 mM BS3 (previously solubilized in PBS as a 50 mM stock). After 30 min at the same temperature, 4× Laemmli Sample Buffer (BioRad #1610747) was added to cross-linked samples and boiled at 95 °C for 5 min. For cross-linking with 1-ethyl-3-(3-(dimethylamino)­propyl) carbodiimide hydrochloride (EDAC), purified His-RAD51­[F86E, A89E] at the same concentration reported above (24 μM (=0.98 mg/mL)) was mixed with a 2-fold stoichiometric excess of biotinylated BRC4 peptide (48 μM, final DMSO concentration lower than 5%). The mixture was incubated for 1 h at 20 °C to allow for complex formation and then cross-linked with a final concentration of 0.2% (w/v) EDAC (previously solubilized in DMSO as a 10% w/v stock) for 1 h at the same temperature. 4× Laemmli Sample Buffer (BioRad #1610747) was then added to cross-linked samples and boiled at 95 °C for 5 min.

### Coomassie Blue and Western Blot Analyses on Cross-Linked Samples

The efficiency of cross-linking reactions was evaluated through Coomassie blue staining and Western blot (WB) analysis. Prepared cross-linked samples were resolved using a 4–15% SDS-PAGE gel (Criterion TGX Precast Midi Protein Gel), which was stained using page blue protein staining solution (Thermo Fisher, 24620) according to manufacturer protocols. Images were acquired using a Gel Doc EZ Imager (Biorad). For WB, an amount equivalent to 50 ng of His-RAD51­[F86E, A89E] and 5 ng of bioBRC4 were loaded. The sample was run on a 12% SDS-PAGE gel (Criterion TGX Precast Midi Protein Gel) and then electrophoretically transferred to TransBlot Turbo nitrocellulose membranes (Midi size, Biorad) using a Transblot Turbo apparatus (Biorad, set at 25 V, 1.0A, 30 min). Membranes were blocked for 1 h at room temperature in 5% Milk TBS-T and, after one wash with TBS-T, were incubated for 1 h at room temperature with Streptavidin HRP to detect the biotin moiety of bioBRC4 (1:3000 dilution in 3% BSA-TBS-T). After three washes in TBS-T, chemiluminescence was detected using the Clarity Western ECL substrate (Biorad, #1705061), and images were recorded using a ChemiDoc MP Imaging System (Biorad).

### LC–MS Analysis

Cross-linking samples in Laemmli Sample Buffer were digested following the *S*-Trap protocol^19^ (Protifi) with minor adaptations and purified using solid-phase extraction. Briefly, a volume of sample containing 100 μg of proteins was diluted to a final volume of 100 μL with 50 mM ammonium bicarbonate (ABC), and proteins were reduced by the addition of 4 μL of TCEP for 30 min at 37 °C with mild agitation. Cysteine alkylation was performed with 8 μL of iodoacetamide (IAA) for 30 min in the dark with mild agitation. The samples were then acidified with 12 μL of 12% phosphoric acid, diluted with 750 μL of the S-Trap binding buffer [1 M triethylammonium bicarbonate (TEAB) buffer and methanol (10:90)], and transferred to *S*-Trap mini columns (Protifi). SDS removal was conducted by three washing cycles with 400 μL of *S*-Trap binding buffer prior to digestion. MS-grade trypsin (Serva) was added in an enzyme to protein ratio of 1:50 using 125 μL of digestion buffer (50 mM ABC, pH 8.5) as media, and the column was incubated overnight at 37 °C with light agitation. Peptides were eluted stepwise in 80 μL of 50 mM ABC, 0.2% formic acid (FA), and 50% acetonitrile (ACN). The pooled fractions were diluted 1:1 with 0.1% trifluoroacetic acid (TFA) and desalted with C18-SPE cartridges (Biotage). After equilibration with 2 mL of ACN, 1 mL of 50% ACN/1% acetic acid, and 2 mL of 0.1% TFA, the samples were loaded onto the cartridge, washed with 2 mL of 0.1% TFA, and eluted with 1 mL of 80% ACN/0.1% TFA. The eluted fractions were dried using an Eppendorf concentrator (Eppendorf) and stored at −20 °C before analysis. Dried peptides were reconstituted in 5% ACN and 0.1% FA. Peptides were loaded onto an Acclaim PepMap C18 capillary trapping column (particle size 3 μm, *L* = 20 mm) and separated on a ReproSil C18-PepSep analytical column (particle size = 1.9 μm, ID = 75 μm, *L* = 25 cm, Bruker Corporation, Billerica, USA) using a nano-HPLC (Dionex U3000 RSLCnano) at a temperature of 55 °C. Trapping was carried out for 6 min with a flow rate of 6 μL/min using a loading buffer composed of 0.05% trifluoroacetic acid in H_2_O. Peptides were separated by a gradient of water (buffer A: 100% H_2_O and 0.1% FA) and acetonitrile (buffer B: 80% ACN, 20% H_2_O, and 0.1% FA) with a constant flow rate of 400 nL/min. The gradient went from 4 to 48% buffer B in 45 min. All solvents were LC–MS grade and purchased from Riedel-de Häen/Honeywell (Seelze, Germany). Eluting peptides were analyzed in data-dependent acquisition mode on an Orbitrap Eclipse mass spectrometer (Thermo Fisher Scientific) coupled to nano-HPLC by a Nano Flex ESI source. MS1 survey scans were acquired over a scan range of 350–1400 mass-to-charge ratio (*m*/*z*) in the Orbitrap detector (resolution = 120k, automatic gain control (AGC) = 2e5, and maximum injection time: 50 ms). Sequence information was acquired by a “ddMS2 OT HCD” MS2 method with a fixed cycle time of 5 s for the MS/MS scans. MS2 scans were generated from the most abundant precursors with a minimum intensity of 5e3 and charge states from two to eight. Selected precursors were isolated in the quadrupole by using a 1.4 Da window and fragmented by using higher-energy collisional dissociation at 30% normalized collision energy. For Orbitrap MS2, an AGC of 5e4 and a maximum injection time of 54 ms were used (resolution = 30k). Dynamic exclusion was set to 30 s with a mass tolerance of 10 ppm. Each sample was measured in duplicate LC–MS/MS runs. MS raw data were processed using the MaxQuant software^20^ (v2.6.5.0) with customized parameters for the Andromeda search engine. Spectra were matched to a FASTA file containing the BRCA2 and RAD51 sequences downloaded from UniProtKB (October 2023), a contaminant and decoy database. The RAD51 sequence was modified to include the His-Tag on the *N*-terminus (MGSSHHHHHHSSGLVPRGSHMLEDP-). A minimum tryptic peptide length of seven amino acids and a maximum of two missed cleavage sites were set. The cross-linker BS3 was chosen from the default list of available cross-linkers. Precursor mass tolerance was set to 4.5 ppm, and fragment ion tolerance was set to 20 ppm, with a static modification (carbamidomethylation) for cysteine residues. Acetylation on the protein *N*-terminus and oxidation of methionine residues were included as variable modifications. A false discovery rate below 1% was applied at cross-link, peptide, and modification levels. Search results were imported to xiVIEW^21^ for subsequent analysis. After stringent manual curation of the spectra, which is a standard practice during the data processing stage, only interprotein cross-links with spectral scores above 30 were selected for fitting on the structures. Cross-links were fitted on the His-Tag-RAD51 and BRC4 and on the untagged RAD51 and BRC4 structures, generated through Alphafold 2.3, using xiVIEW.

### AlphaFold Predictions

All predictions reported in this work were generated through an AlphaFold 2.3.2 run as a singularity container. The model preset used for prediction was set to multimer, enabling Amber relaxation, which resolves remaining structural violations and clashes in the predicted structure through an iterative restrained energy minimization via gradient descent with the Amber ff99SB force field,^22^ only for the highest ranked model. Database preset was set to full database (full_dbs databases: bfd, mgnify (mgy_clusters_2022_05), pdb_mmcif, pdb_seqres, pdb70, Uniref 30_2023_02, uniprot, uniref90). Graphs of multiple sequence alignment coverage and sequence similarity, predicted local distance difference test, and predicted aligned error were generated through Python scripts adapted from https://raw.githubusercontent.com/jasperzuallaert/VIBFold/main/visualize_alphafold_resultr.py and https://raw.githubusercontent.com/busrasavas/AFanalysis/main/AFanalysis.py

### SAXS Spectra Calculation

Experimental observables can be predicted from structures through forward models, i.e., equations relating measured quantities to structural features. Herein, the calculation of SAXS spectra from both AlphaFold2 and MD-sampled structures was performed with the PLUMED library,^23,24^ version 2.9, via the integrative structural and dynamical biology (ISDB) module.^25^ In particular, the SAXS spectrum of a given three-dimensional structure can be calculated according to the following equation

1 $$I\left(q\right)=\sum_{i=1}^{N}\sum_{j=1}^{N}f_{i}\left(q\right)f_{j}\left(q\right)\frac{\sin\left(qr_{ij}\right)}{qr_{ij}}$$

where the scattered intensity *I* at a certain value of the momentum transfer *q* is obtained from the pairwise distance *r*_ij_ between all pairs of the *N* atoms in the biomolecules and the corresponding atomic scattering factors *f*_*i*_(*q*) and *f*_*j*_(*q*).

Calculating the pairwise distance between each pair of atoms in a macromolecular system at an atomistic resolution is computationally demanding. This becomes particularly critical if the calculation has to be repeated many times, e.g., on a large structural ensemble or on-the-fly during MD simulations. For this reason, a coarse-grained representation can improve the efficiency of the calculation. Specifically, the atomistic representation can be simplified by grouping together a given number of atoms into pseudoatoms, which are also called beads. This results in a system representation comprising *M* beads, with typically *M* ≪ *N,* and the computed scattering intensity can be expressed as

2 $$I\left(q\right)=\sum_{i=1}^{M}\sum_{j=1}^{M}F_{i}\left(q\right)F_{j}\left(q\right)\frac{\sin\left(qR_{ij}\right)}{qR_{ij}}$$

where *R*_ij_ is the distance between pairs of beads. Notably, this implies that scattering factors *F* associated with the pseudoatoms are available, i.e., have been purposely parametrized. Most interestingly, a great advantage of this strategy is that it can be exploited in a hybrid coarse-grain/all-atom fashion. Specifically, the MD simulations can be conducted at a fully atomistic resolution, while resorting to a coarse-grained representation for the sole purpose of efficiently computing the SAXS spectra (hySAXS scheme).

The MARTINI force field is one of the most popular coarse-grained models for biomolecules.^26^ In such a paradigm, amino acid residues are mapped into a varying number of beads, ranging from 1 (e.g., alanine) to 5 (e.g., tryptophane). Scattering factors of MARTINI beads have been parametrized for both protein and nucleic acid systems, allowing for hySAXS scheme simulations exploiting MARTINI as the coarse-grained model (hereafter referred to as MT-hySAXS). Recently, an alternative coarse-grained model for SAXS spectra calculation was introduced, named Single-Bead.^27,28^ In the protein context, this representation replaces each amino acid with a single bead, hence the name. Despite being more simplified than the MARTINI representation, thus further improving the efficiency of SAXS spectra calculation, this model retains comparable accuracy, particularly for *q* values lower than 0.3 A^–1^.^28^ Remarkably, the single-bead approach (SB-hySAXS) features a solvent layer contribution term^28^ in the definition of scattering factor *F* that allows accounting for the effect of solvation on solvent-exposed atoms in the biomolecule.

### Steered MD Simulations

All MD simulations were carried out at the atomistic level using the GROMACS MD engine,^29^ using the Amber ff19SB force field^30^ for the proteins, and the OPC model for water molecules.^31^ The full structure of the RAD51–BRC4 complex predicted with AlphaFold2 was inserted in a dodecahedron-shaped box, with edges 15 Å from the biomolecules. The box was then filled with OPC waters, and the system was neutralized and brought to physiological ionic concentration (0.15 M) with NaCl using Joung and Cheatham ions.^32^ The system was energy-minimized using the steepest descent method and subsequently equilibrated via a 1.2 ns simulation in the *NVT* ensemble with position restraints of 239 kcal/mol on protein heavy atoms (of which 400 ps was conducted at 100 K, 400 ps at 200 K, and the last 400 ps at 300 K) and 800 ps in the *NPT* ensemble (of which, 400 ps was conducted with protein heavy atoms restraints, while the last 400 ps only featured α carbon atoms restraints) using the *V*-rescale thermostat^33^ with time constant 0.1 for temperature control and *C*-rescale barostat^34^ with time constant of 0.1 for pressure control.

Steered MD (sMD) simulations were carried out via the MOVINGRESTRAINTS directive in PLUMED 2.9.^23,24^ Three replicates of 10 ns each were performed using the Martini coarse grain representation for SAXS spectra calculations and three replicates of 10 ns using the single-bead model.

A set of 19 SAXS intensities computed at different values of the momentum transfer *q*, in the range 0.00–0.30 Å^–1^, was used as a collective variable (CV). During the 10 ns of sMD, the positions of the harmonic restraints were linearly interpolated from the values of the SAXS intensities computed on the initial structure, i.e., the AlphaFold2 model, to the experimental ones. The SAXS experimental spectrum for the RAD51–BRC4 complex was taken from the Small Angle Scattering Biological Data Bank (SASBDB), under accession code SASDQT9. To reduce the influence of experimental noise, the experimental values were taken after a 51-point running average was performed on the experimental SAXS spectrum. The force constant was kept constant at a value of 10^6^ kJ/(mol·a.u.^2^) for the entire simulation.

### Metadynamics Simulation

A 100 ns well-tempered metadynamics (metad)^35^ run in the *NVT* ensemble was carried out via PLUMED 2.9, using the radius of gyration as CV. Gaussians of width 0.05 Å and height 0.500 kcal/mol were deposited every 500 steps, using a bias factor of 10. The distances between Cα atoms of the four pairs of cross-linked lysine residues, as indicated by the XL-MS experiments, were restrained through a flat-bottom restraining potential, as implemented in the UPPER_WALLS PLUMED directive, positioned at a value of 30 Å^36^ with a force constant of 23.90 kcal/(mol·Å^2^). Specifically, the restrained distances were the ones between RAD51’s K59 and BRC4’s K24, RAD51’s K59 and BRC4’s K29, RAD51’s K65 and BRC4’s K29, and RAD51’s K71 and BRC4’s K29. Metad weights were computed a posteriori by using the final bias.

### Maximum Entropy Reweighting

The maximum entropy principle can be used to reweight ensembles,^14,37,38^ ensuring consistency with experimental data while inducing the least possible modification to the original distribution (the prior). In our case, the pool of conformations sampled by metad was reweighted to bring the computed average SAXS spectrum into agreement with the available experimental data.

Through this procedure, new weights *w*_*t*_ are assigned to each configuration *x*_*t*_ from the original ensemble comprising *N*_s_ structures (i.e., the frames in the MD trajectory), according to

3 $$w_{t}=e^{-\sum_{i}\lambda_{i}^{*}\cdot s_{i}\left(x_{t}\right)}$$

In this expression, *s*_*i*_(*x*_*t*_) is the value of the *i*th observable (here, the *i*th intensity of a SAXS spectrum) computed for structure *x*_*t*_, and λ_i_^*^ is the corresponding Lagrangian multiplier that minimizes the Lagrangian function

$$\Gamma \left(\lambda \right)=\ln\left(\frac{1}{N_{s}}\overset{Ns}{\underset{t=1}{\sum }}e^{-\sum_{i}\lambda_{i}\cdot s_{i}\left(x_{t}\right)}\right)+\sum_{i}\lambda_{i}\cdot s_{i}^{\exp}+\Gamma_{err}\left(\lambda \right)$$ 4

This minimization is the equivalent of Shannon’s Entropy maximization.^39^ As a result, the (weighted) average of the computed observables *s* along the reweighted trajectory is constrained to the measured experimental value *s* ^exp^.

The last term in the equation is introduced to model experimental error, acting as a regularization term to reduce overfitting, and can be defined as follows

5 $$\Gamma_{err}\left(\lambda \right)=\frac{1}{2}\sum_{i}\lambda_{i}^{2}\sigma_{i}^{2}$$

where a different σ_*i*_ value can be specified for each observable *s*_*i*_.

Herein, we used a prior ensemble generated via metad, characterized by uneven weights associated with the metad bias. In this case, the above formulations can be rewritten as

6 $$w_{t}=e^{-\sum_{i}\lambda_{i}^{*}\cdot s_{i}\left(x_{t}\right)+V_{b}\left(x_{t}\right)/k_{B}T}$$

7 $$\Gamma \left(\lambda \right)=\ln\left(\frac{1}{N_{s}}\sum_{t=1}^{N_{s}}e^{-\sum_{i}\lambda_{i}\cdot s_{i}\left(x_{t}\right)+V_{b}\left(x_{t}\right)/k_{B}T}\right)+\sum_{i}\lambda_{i}\cdot s_{i}^{\exp}+\Gamma_{err}\left(\lambda \right)$$

where *V*_b_(*x*_*t*_) is the metad bias at the end of the simulation recomputed for the coordinates of the *x*_*t*_^th^ frame, and *k*_B_ *T* is the thermal energy. Herein, minimization was conducted through the optimize module in SciPy^40^ using the BFGS (Broyden–Fletcher–Goldfarb–Shanno) method,^41–43^ and for simplicity, we applied the same σ value to all observables, i.e., to all points in the spectrum.

Typically, only a fraction of the structures from the prior ensemble contribute effectively to the reweighted ensemble. The number of such structures that carry significant weight can be approximately estimated by calculating the Kish effective sample size

8 $$K=\frac{{\left(\sum_{t=1}^{N_{s}}w_{t}\right)}^{2}}{\sum_{t=1}^{N_{s}}w_{t}^{2}}$$

A parameter that is typically employed to assess overfitting in the context of SAXS spectra is the reduced χ^2^

9 $$\chi^{2}=\frac{1}{m-1}\sum_{i=1}^{m}{\left(\frac{I_{fit}\left(q_{i}\right)-I_{\exp}\left(q_{i}\right)}{SE\left(I\left(q_{i}\right)\right)}\right)}^{2}$$

where *I*_fit_(*q*_*i*_) and *I*_exp_(*q*_*i*_) are the predicted (i.e., from the reweighted ensemble) and experimental intensities, and SE­(*I*(*q*_*i*_)) is the standard error on the intensities at each of the *m* values *q*_*i*_ of the SAXS spectrum. χ^2^ values close to 0 indicate overfitting, while values around 1 indicate optimal fitting of the experimental data. Here, SE­(*I*(*q*_*i*_)) included both the experimental and predicted uncertainties after error propagation.

### Trajectory Analysis

The trajectory obtained from the metad simulation was analyzed through principal component analysis carried out on the Cartesian coordinates of the system’s α carbons after aligning the snapshots on the α carbons of the *C*-ter domain. The first 5 PCs, explaining a cumulative variance of 93%, were used to compute a distance matrix on which we performed a weighted cluster analysis using the quality threshold (QT) algorithm, with a cutoff of 3 and the maxent weights. The cluster analysis, performed using the py-bussilab Python package (https://github.com/bussilab/py-bussilab), was done separately on compact and extended structures, using an *R*_g_ value of 2.47 Å to discriminate between the two groups. All reported statistical errors were computed through standard bootstrapping with 400 iterations after dividing the snapshots from the metad-generated trajectory into 10 blocks.

The ensemble of conformations was summarized and visualized by constructing a conformational space network where the representative structure of each cluster served as a node. Edges in the network were assigned using a distance cutoff of 5, whereas the size of the nodes is proportional to the maxent weights. To better visualize the most populated clusters in the network, the weights were rescaled using a logistic function. The Pyvis-0.1.3.1 software^44^ was used to represent the network, employing the BarnesHut^45^ layout algorithm.

The contact analysis on the reweighted ensemble was performed using the compute_contacts module in MDTraj,^46^ using the closest-heavy scheme to compute minimum distances between pairs of residues. To define a contact, we employed a distance cutoff of 5 Å. The weighted average of the number of contacts was computed using the weights of the maxent-reweighted ensemble. To highlight the residues mainly involved in the interfacial interaction, we used a 2.5% probability threshold in the reweighted ensemble, and any contacts exceeding this value were deemed as persistent.

## Results

### Probing the Interaction between RAD51’s *N*-ter and BRC4 by Combining AlphaFold2 and XL-MS

As no high-resolution structure of the full RAD51 in complex with BRC4 is available, we generated an AlphaFold2 model, which would provide an initial reasonable guess of the complex structure.^22,47,48^ In line with our previous findings, we observed that AlphaFold predicted a conformational rearrangement of the RAD51 *N*-ter to allow for the BRC4 peptide binding^4,9,11^ (Figure S1). Moreover, we noted the presence of a network of polar contacts stabilizing the interaction of the BRC4 *C*-ter with the RAD51 *N*-ter, thus suggesting another important interface for the peptide binding, as also highlighted by a previous work^49^ (Figure S2). To test the existence of this putative interaction between BRC4 and the protein *N*-ter, we decided to apply cross-linking mass spectrometry (XL-MS). Indeed, this technique would provide us with useful structural information, overcoming the difficulties of achieving a 3D crystal structure, as we observed the presence of different lysine residues at the predicted interface between the BRC4 peptide and the RAD51 *N*-ter (Figure S2).

Considering the small size of the BRC4 peptide, we engineered a modified version harboring a biotin at the *N*-ter (bioBRC4), which we utilized to reconstitute in vitro the RAD51–BRC4 complex by mixing bioBRC4 with the monomeric His-RAD51­[F86E, A89E] (hereafter referred to as “monomeric RAD51”). This would easily allow for the identification of cross-links by performing a WB analysis tracking the bioBRC4 through Streptavidin coupled to Horse Radish Peroxidase (Streptavidin-HRP). We initially confirmed the binding of bioBRC4 to monomeric RAD51 by exploiting two orthogonal methods, SLS and BLI analyses (Figure S3).

Having assessed that the modification of the peptide did not negatively affect its binding to monomeric RAD51, we cross-linked the reconstituted bioBRC4-monomeric RAD51 complex, using both 1-ethyl-3-(3-(dimethylamino)­propyl)­carbodiimide hydrochloride (EDAC) and bis­(sulfosuccinimidyl)­suberate (BS3) with spacer lengths of 0 and 11.4 Å, respectively. Initially, Coomassie blue-stained SDS-Page gel analyses suggested that BS3 cross-linked bioBRC4 to the monomeric RAD51 more effectively than EDAC. We then confirmed our observation by WB analysis in which the bioBRC4 signal was clearly shifted to a molecular weight between 37 and 50 kDa, matching a cross-linked RAD51–BRC4 complex (Figure S4). At this stage, we analyzed the cross-linked complex using LC–MS/MS on an Eclipse instrument. XL-MS analysis led to the identification of four high-quality cross-links between BRC4 and RAD51 (Figures 2A, S5, and Table 1). Specifically, we observed that Lys1536 and Lys1541, in proximity of the BRC4 LFDE domain, were found to be cross-linked with Lys59, Lys65, and Lys71 located on the RAD51 *N*-ter in a region encompassing a cluster of alpha-helices, which support RAD51 interaction with the DNA (Figures 2A, S5, and Table 1).^50^

**Figure 2:**
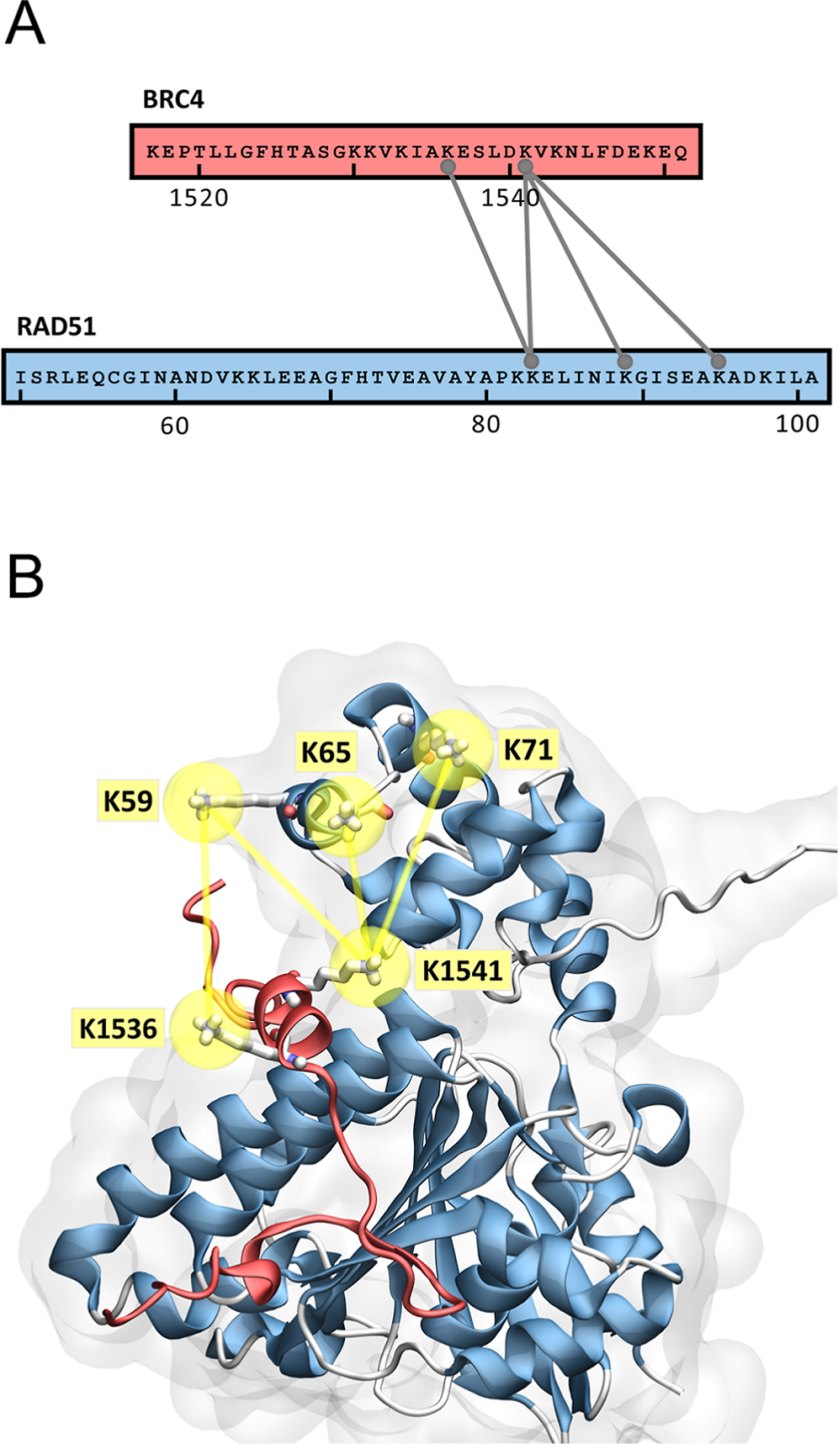
XL-MS data show cross-links between RAD51 and BRC4. (A) Sequence overview of the BRC4 repeat of BRCA2 and His-tagged RAD51 illustrating the detected interprotein cross-links filtered for MS quality score >30 and Cα–Cα distances of 10–35 Å. Connecting lines point toward the cross-linked residues. (B) Mapping of the identified cross-links in the AlphaFold2 prediction of the RAD51–BRC4 complex.

**Table 1:** Detected Inter-protein Crosslinks and Their Measured Cα–Cα Distances in the AlphaFold2 Prediction of the His-RAD51/BRC4 Complex (+His), Exploited for XL-MS Experiments, and the RAD51/BRC4 Complex (−His), Utilized in SAXS Experiments

position in BRCA2	position in RAD51 (+His)	Cα–Cα distance (Å) (+His)	position in RAD51 (−His)	Cα–Cα distance (Å) (−His)
1541	89	16.80	65	16.04
1541	95	21.24	71	21.11
1541	83	12.39	59	21.76
1536	83	18.84	59	30.38

This result highlighted that the BRC4 *C*-terminus is crucial to allow for the binding of the peptide to RAD51, since it directly interacts with the RAD51 *N*-ter, displacing it. Then, we aimed to verify the compliance with cross-linking data of His-RAD51/BRC4 and RAD51/BRC4 AlphaFold2 models, respectively, employed for XL-MS or SAXS experiments (Figures 2, S1, S2, S6, S7). Notably, in both predictions, the identified pairs of cross-linked lysine residues displayed Cα–Cα distance compatible with the experimental XL-MS results (Table 1) and within the limits of typical distance constraints, further corroborating our initial observation, made on the RAD51–BRC4 AlphaFold2 model, that a hydrogen bond network amidst the BRC4 *C*-terminus and the RAD51 *N*-term exists.^36^ Additional validation of the generated AlphaFold2 predictions was provided by distances of identified intra-RAD51 cross-links. Indeed, the majority of identified cross-links matched permissive distances in the predicted structure, with only two exhibiting long distances >45 Å (Figure S8). While we cannot a priori exclude that the latter are experimental XL-MS false identifications, they may also derive from RAD51 *N*-ter flexibility in solution when BRC4 is bound.^11^ Indeed, the conformational rearrangements of this domain could potentially bring the lysine residues in closer proximity to each other, thus allowing for the cross-linking reaction by BS3.

### Including the Solvent Contribution is Necessary for Generating a SAXS-Consistent Single-Structure Model of the RAD51–BRC4 Complex

Having validated the RAD51–BRC4 AlphaFold2 prediction by XL-MS, we then compared its computed SAXS spectrum with the experimental one recently determined for the RAD51–BRC4 complex in solution, already available in SASBDB.^11^ Nevertheless, we observed a significant discrepancy between the initial AlphaFold2 guess and experimental SAXS data (Figure 3A), as the predicted spectrum indicated an overly compact configuration compared to experiments.^51^

**Figure 3:**
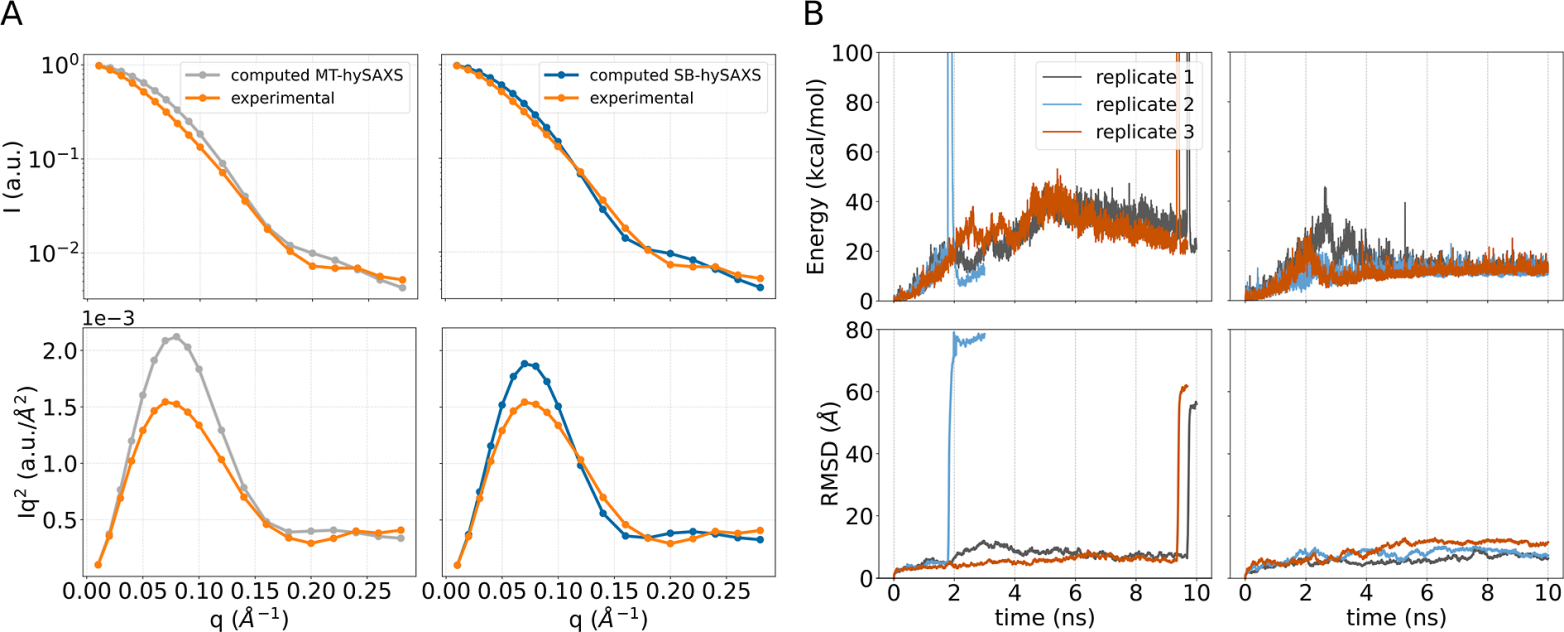
A) Logarithmic scale (top) and Kratky (bottom) plots of the SAXS spectrum computed on the AlphaFold model without (MT-hySAXS scheme, left) and with (SB-hySAXS scheme, right) solvent contribution, compared with the experimentally measured SAXS spectrum of the complex. (B) Timeseries of the sMD bias (top) and BRC4 heavy atoms RMSD after alignment on the *C*-ter (bottom), carried out without (left) and with (right) solvent contribution.

To guide the initial guess toward a configuration in better agreement with experimental data, we took advantage of steered MD (sMD) simulations. sMD is an enhanced sampling method in which the exploration of complex biomolecular processes along a predefined CV is improved by using a time-dependent biasing potential. Typically, the biasing potential takes the form of a harmonic restraint that moves at a constant velocity during the simulation, driving the system toward a target value of the CV. Here, the system was driven toward the target state, defined by the experimental SAXS spectrum, through a hybrid all-atom/coarse-grain scheme (see Methods) over the course of 10 ns-long sMD simulations. Unexpectedly, in all three replicates, the simulation invariably resulted in the detachment of the BRC4 repeat from RAD51, as indicated by a rapid rise in the energy and quantified by a marked increase in the RMSD of the complex (Figure 3B, left top and bottom panels, respectively). Notably, this behavior is in contrast with the experimental SAXS data, which refer to the formed complex, as we have formerly demonstrated.^11^ We note that this set of calculations relied on a forward model that does not take into account the contribution of the protein’s solvation layer to the SAXS spectra (MT-hySAXS scheme). Remarkably, repeating the procedure by taking into account solvation effects (SB-hySAXS scheme) led to the preservation of the RAD51–BRC4 complex, as indicated by the absence of sudden rises in the energy as well as in the RMSD (Figure 3B, right panels). Importantly, most of the conformational rearrangements required to match the experimental SAXS spectrum involved *N*-ter (Figures S9 and S10). We stress that this result was achieved by the simulations without any prior information about flexible regions in RAD51, nor by introducing restraints on the BRC4 repeat to prevent detachment. Thus, in the effort to satisfy the experimental SAXS data, the system was able to naturally adapt based on its physical properties. We refer to the configurations attained by the system at the target state of the sMD simulation, obtained using the SB-hySAXS scheme, as single-structure models. Three replicates of sMD with the SB-hySAXS scheme resulted in comparable single structure models (Figures S9 and S10). These models were instrumental for providing a SAXS-consistent initial state for the subsequent XL-MS-informed metadynamics (metad) simulations. Here, we used the single-structure model from the first replicate of the SB-hySAXS sMD for the subsequent stage.

### XL-MS-Informed Simulations Enable Determination of a SAXS-Compliant Conformational Ensemble of the RAD51–BRC4 Complex

As previously suggested, binding of the BRC4 peptide to RAD51 could trigger a conformational rearrangement of RAD51’s *N*-ter, which could behave as an intrinsically disordered domain.^11^ Given this scenario, an ensemble of conformations is better suited to achieve a more realistic description of the system.^16,52^ Therefore, we aimed to identify a conformational ensemble of structures that would be compatible with the experimental SAXS data.^11^ To this end, we first generated a heterogeneous ensemble of RAD51–BRC4 configurations; then, the population weights were refined through a reweighting procedure in order to match experimental data. For the first stage, MD simulations did not include any information about the experimental SAXS spectrum except for the use of the SAXS-consistent single-structure of the RAD51–BRC4 complex obtained from the first replicate of the sMD simulations as a starting configuration. Specifically, we carried out metad simulations, an enhanced sampling method that allows for a comprehensive exploration of the configurational space along predefined CVs by applying a time-dependent Gaussian-shaped bias potential.^35^ Taking the inverse of the total bias potential at the end of the metad simulation enables the reconstruction of the corresponding free energy profile. In this case, to promote the exploration of RAD51–BRC4 configurations with varying degrees of structural compactness, we used the radius of gyration of the complex as a CV (Figures S11A,B). Additionally, metad simulations were integrated with information from the XL-MS experimental data in the form of distance restraints between RAD51’s *N*-ter and the BRC4 repeat (Figure S11C). Including such information avoided sampling of irrelevant states that would be incompatible with the maximum distances, as suggested by the XL-MS data. This favored a more effective exploration of the system’s configurational space and, in turn, optimized the efficiency of the simulation. Therefore, the RAD51–BRC4 structures produced in this way were used as a prior ensemble for the subsequent reweighting procedure according to the maximum entropy principle, using the experimental SAXS spectrum as the ground truth. To avoid overfitting, we introduced a regularization term in the procedure, chosen to result in an optimal value of the reduced χ^2^ of about 1 between reweighted and experimental SAXS spectra (see Methods), as typically recommended for a model that adequately describes the experimental data.^53–56^ As a result, we were able to identify an ensemble of structures with a calculated SAXS spectrum in agreement with the experimental one within statistical error (Figures 4, and S12). This reweighting stage resulted in a Kish effective sample size of 7391, out of the 20,001 structures used in the analysis. This indicates that a significant portion of structures from the prior ensemble, derived from the metad simulation, were retained in the maxent-reweighted one and effectively contributed to the final computed SAXS spectrum. We note that compatible results can be obtained using a smaller number of frames, provided that they preserve the diversity of the conformational space explored. Herein, we decided to retain the maximum amount of structural information possible and used cluster analysis to facilitate the interpretation of the results.

**Figure 4:**
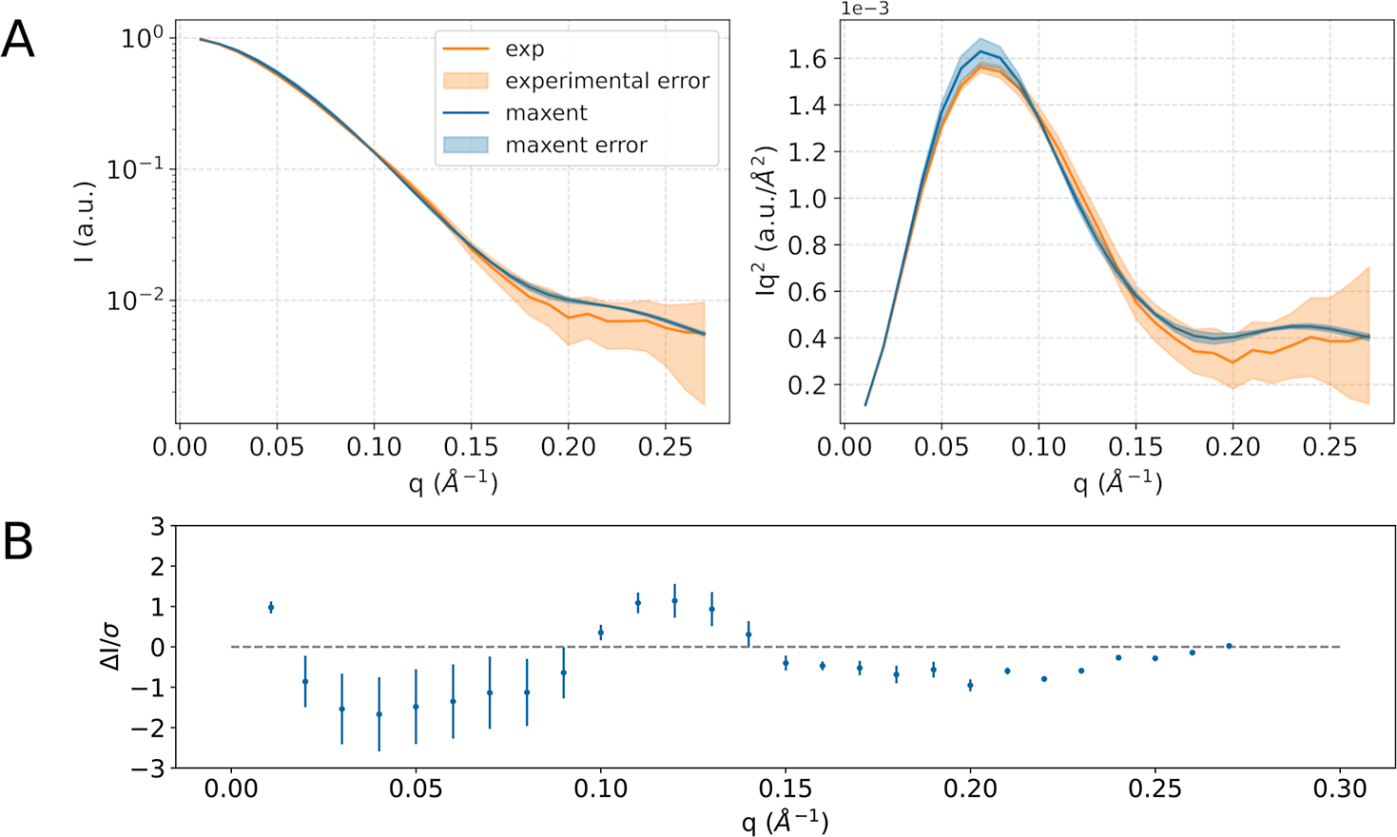
A) Comparison of the reweighted SAXS spectrum through maxent with the experimental one, in logarithmic scale (left) and Kratky form (right), for the RAD51–BRC4 complex. (B) Residual plot of the computed spectrum with respect to the experimental one.

### Charged Residues at the *N*-ter-BRC4 Interface Bridge Compact and Extended Structures in the Reweighted Ensemble

A closer inspection of the reweighted ensemble revealed the presence of compact and extended conformations with low and high values of the radius of gyration, respectively. The prior ensemble from metad showed a deeper energy well centered at *R*_g_ ≈ 23 Å, while maxent reweighting shifted the population by assigning higher weights to the more elongated structures in the shallower energy well at *R*_g_ ≈ 29 Å (Figure 5). The maxent reweighting, therefore, resulted in a more balanced ensemble, with 65% extended and 35% compact structures. In contrast, the metad population was almost entirely composed of compact conformations (∼99%).

**Figure 5:**
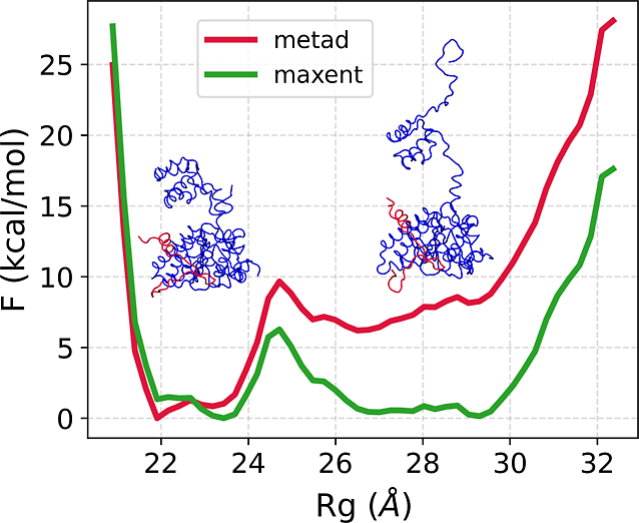
Free energy as a function of the radius of gyration for the metad ensemble (red) and the reweighted ensemble via maxent (green).

To better appreciate the observed maxent conformational heterogeneity of the RAD51–BRC4 complex in solution, we decided to represent it as a conformational space network as reported in Figure 6. Nodes in the network represent distinct clusters obtained from the metad simulations, with their size proportional to the corresponding maxent weights (Table S1). The presence of edges indicates substantial similarity among different cluster representatives. Therefore, the network topology reflects the composition of the reconstructed conformational ensemble, allowing us to appreciate the distinctive features of the compact and extended subsets of structures (shown as orange and light blue nodes in Figure 6, respectively) and their mutual relationships.

**Figure 6:**
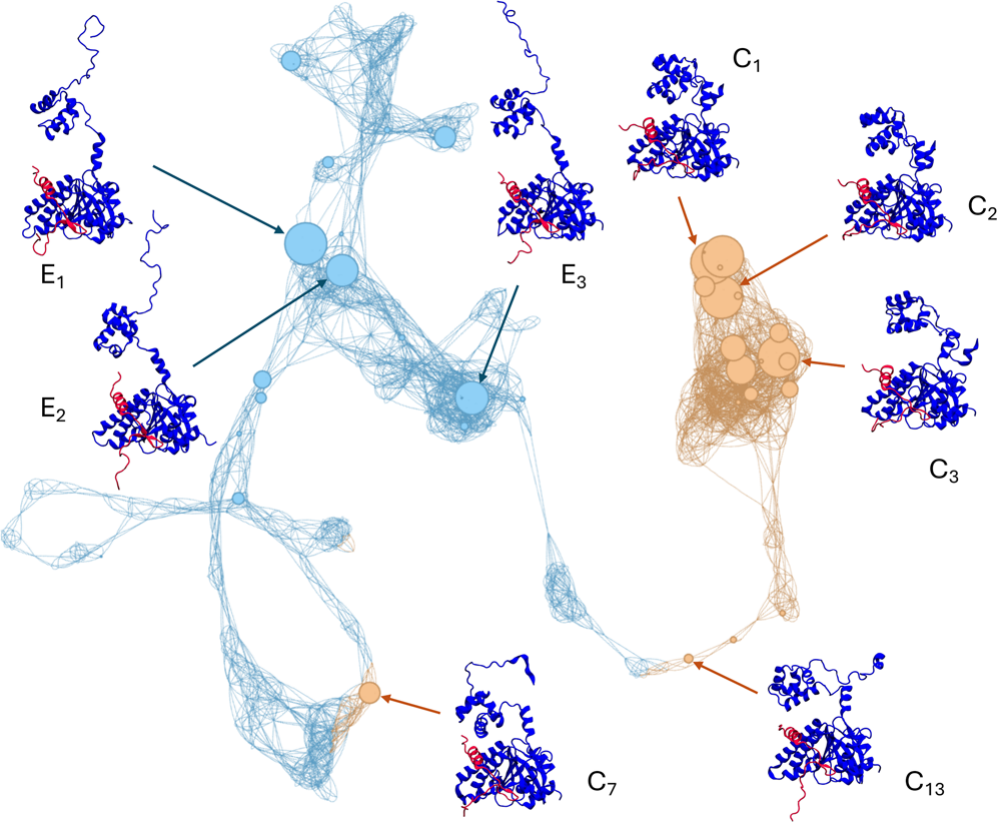
Conformational space network showing the reconstructed RAD51–BRC4 ensemble. Each node represents a cluster, whose size is proportional to the maxent reweighted population. Nodes are colored based on the degree of structural compactness, as indicated by the reweighted free energy profile in Figure 5 (orange for compact, light blue for extended). The representative structures of selected nodes, corresponding to either the most populated clusters or regions with peculiar network topology, are shown to highlight the conformational heterogeneity of the reconstructed ensemble.

Most compact structures are located in a dense, highly interconnected region of the plot, highlighting a striking conformational similarity among the representative members of the different clusters. Nodes *C*_1_, *C*_2_, and *C*_3_ represent compact states with high statistical weight, showing an increasing and progressive separation between RAD51’s *N*-ter and BRC4. Conversely, the regions of the network representing extended structures are more scattered, reflecting greater dissimilarity due to a higher conformational freedom associated with the full detachment of RAD51’s *N*-ter and increased disorder. Nodes *E*_1_, *E*_2_, and *E*_3_ are examples of clusters with high statistical weight belonging to the extended subset of conformations. Additionally, node *C*_13_ emerges as an intermediate structure connecting the compact and extended states, showing a substantially compact conformation with a partly disordered RAD51’s *N*-ter. While marginal in statistical weight, isolated compact states also appear in different regions of the network, with node *C*_7_ being the most populated. As revealed by the conformational space network, the difference in structural compactness is mainly governed by configurational rearrangements of the *N*-ter domain with respect to the complex between BRC4 and RAD51’s *C*-ter (Figure 6). This is also reflected by diverse distributions of the distances between the cross-linked residues within the different clusters (Figure S13), with generally broader distributions shifted to lower values for the clusters of compact structures.

To gain deeper mechanistic insights into RAD51–BRC4 complex formation, we analyzed the interaction at the interface between BRC4 and RAD51’s *N*-ter in the maxent-reweighted ensemble (Figures 7, and S14). To this end, we performed a contact analysis between BRC4 and *N*-ter, which revealed that the interaction is predominantly mediated by charged and polar residues. In particular, the analysis highlighted the involvement of several charged and polar residues at the interface, including three lysines, three glutamates, one glutamine, and one serine, which formed the most persistent contacts. These findings underscore the notable enrichment of lysine and glutamate side chains in driving the interaction. A few hydrophobic residues, such as one leucine and one alanine, also contributed to the interface. Lys59 of RAD51’s *N*-ter, specifically, emerged as a key contact residue, frequently engaging with Glu38 and Gln39 from the BRC4 repeat. These residues form a localized electrostatic and hydrogen-bonding hotspot at the interface, suggesting that their interaction may act as a molecular anchor stabilizing the compact subset of structures. The spatial distribution and contact frequency of the residues suggest that electrostatic interactions between RAD51’s *N*-ter and BRC4 can have a central role in regulating the complex’s structural dynamics.

**Figure 7:**
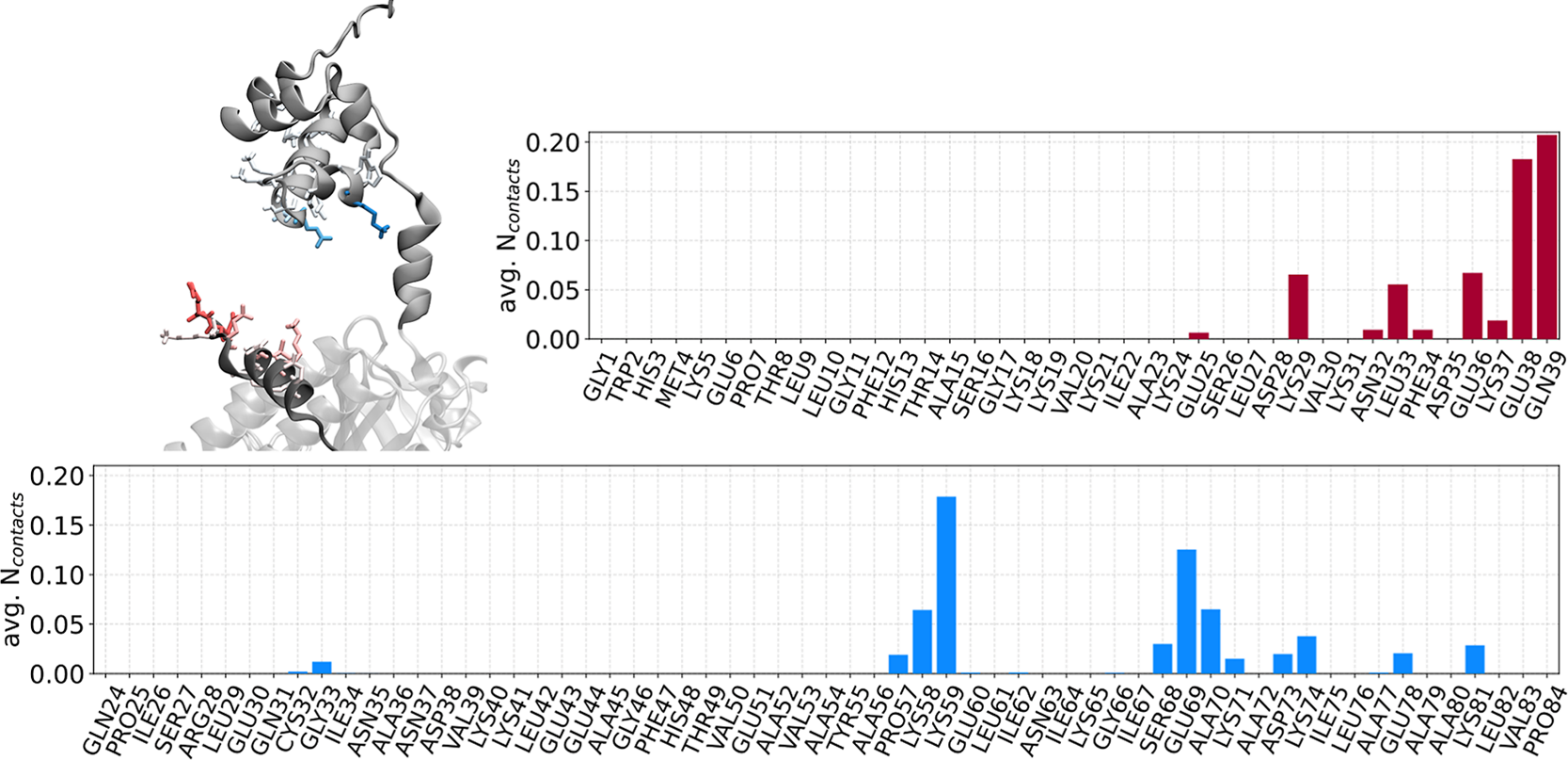
Residue-wise average number of contacts for the structures in the maxent-reweighted ensemble. The analysis focused on contacts for residues from the BRC4 repeat (in red) and RAD51’s *N*-ter (in blue). In the 3D structure, residues with nonzero contacts are displayed in licorice, colored consistently with the plots and with color ranging from white to full solid, indicating low to maximum number of observed contacts, respectively; the *C*-ter, *N*-ter, and BRC4 are displayed in VMD’s NewCartoon representation, colored white, gray, and dark gray, respectively.

## Discussion

In this work, we aimed at providing the first comprehensive reconstruction of the conformational ensemble of the full-length RAD51 protein in complex with the BRC4 repeat, an interaction for which no experimental structure is currently available. To address the challenges posed by the high flexibility of RAD51’s *N*-ter domain, we have complemented advanced MD simulations with newly acquired XL-MS and recently published SAXS data. The XL-MS studies confirmed the interaction between the BRC4 *C*-terminus and the RAD51 *N*-terminal domain, thus providing initial validation of the Alphafold2 model and valuable constraints for further computational studies. Moreover, this result offers key mechanistic insight into how BRC4 interacts with the RAD51 fibrils. In this process, residues located at the BRC4 *C*-terminal domain are crucial as they interact with the RAD51 *N*-ter and promote the detachment of monomers from RAD51 fibril termini.^12^ Nevertheless, although this structure showed lysine pair distances consistent with the XL-MS experimental data, it was incompatible with the experimental SAXS measurement, irrespective of the forward model used to calculate the spectra. Interestingly, including the solvation layer contribution resulted in a predicted spectrum closer to the experimental one, underscoring the significant role of the solvent in SAXS calculations for this system, as also confirmed by the sMD simulations. The resulting single-structure model, now consistent with the experimental SAXS spectrum, proved instrumental in generating a reasonable prior ensemble via subsequent metad simulations incorporating XL-MS restraints. Notably, this initial ensemble of structures, enriched with configurations compatible with experimental data and spanning a wide range of structural compactnesses, enabled successful reweighting via the maximum entropy principle, as demonstrated by the relatively large Kish size. Specifically, to achieve a reconstructed ensemble in agreement with the SAXS experimental data, a larger proportion of extended structures needed to be included and assigned higher weights.

To provide further insights into the RAD51–BRC4 interaction, we examined the reweighted ensemble from a mechanistic standpoint, investigating the possible interactions between residues from the RAD51 *N*-ter domain and the BRC4 repeat. Our contact analysis pinpointed charged residues at the *N*-ter-BRC4 interface, particularly Lys59 from the *N*-ter and Glu38 and Gln39 from the BRC4 repeat. We note that such information was retrieved in a dynamical setting, as the structures were identified from MD simulations. This suggests how the long-range nature of the interaction between charged residues may bridge the transition from compact states to more extended states. Besides providing additional details in the comprehension of RAD51–BRC recognition, this information may be leveraged for the rational design of binders at the BRC4 site modulating RAD51’s activity.

To the best of our knowledge, this is the first report comprehensively characterizing the full conformational ensemble of the RAD51–BRC4 interaction in solution with atomistic resolution, providing insights into critical residues underpinning their complex conformational dynamics. Our study highlights that BRC4 can depolymerize RAD51 fibrils, thanks to the direct interaction of its residues located at the *C*-terminus with the RAD51 *N*-terminal domain. Specifically, this interaction is crucial to drive the RAD51 *N*-terminal domain into the solvent, where it explores multiple conformations. The observed multifaceted behavior of RAD51 *N*-ter is likely necessary to prevent the RAD51 capacity to oligomerize, thus allowing for its translocation in a monomeric form inside the nucleus at the site of DNA damage. RAD51 oligomerization is mediated by two main interfaces: the first one is provided by an ATP molecule placed between two adjacent protomers, while the second one is mediated by a short β-strand in the RAD51 *N*-ter (residues 85-GGFTTATE-91), known as the oligomerization motif, which binds to a central β-sheet of the ATPase domain of the neighboring RAD51 protomer.^57^ Mutations at both F86 and A89 have been shown to significantly impair protomer–protomer affinity and oligomerization.^9,58^ The BRC4 peptide through the FXXA domain (residues 1521-LGFHTASG-1529) mimics the RAD51 oligomerization motif, thus blocking RAD51 from interacting with another RAD51 protomer. Moreover, we observed that charged residues located near the BRC4 LFDE domain (residues 1543-KNLFDEKE-1550) displace the RAD51 *N*-ter, which subsequently explores multiple conformations in solution. Therefore, our work suggests that while the BRC4 FXXA domain sterically hinders the binding of an additional RAD51 monomer, the LFDE domain contributes to maintaining *N*-ter flexibility, thereby significantly impairing the oligomerization motif’s ability to recognize another RAD51 protomer. The combined effect of the BRC4 FXXA and LFDE domains prevents the formation of oligomers, thereby inhibiting ATP binding and further blocking RAD51 oligomerization. Dissecting the mechanistic details of the interaction with the BRC4 repeat is fundamental to understanding the molecular features governing the recognition between RAD51 and the BRCA2 protein. Indeed, considering the close homology of BRC repeats, we envision that the purported mechanism can also be valid for the interaction of other BRC repeats with the RAD51 *N*-ter. Our results offer novel mechanistic insight into the role of BRC repeats in driving RAD51 translocation inside the nucleus, an essential first step in the HR process.^12^ This information is key to elucidating the molecular mechanisms underlying the severe pathological conditions associated with DNA damage repair. In this context, our study provides valuable insights to understand how mutations in residues essential for the BRC4-RAD51 *N*-ter interaction can impact RAD51 recruitment, offering key information for developing rational therapeutic strategies targeting the BRCA2-RAD51 interaction.

## Supplementary Material



## Data Availability

SAXS data supporting this study are openly available in SASBDB (https://www.sasbdb.org/) with reference number SASDQT9—Monomeric DNA repair protein RAD51 homologue 1 double mutant [F86E, A89E] in complex with fourth BRC repeat (BRC4) (66). The mass spectrometry proteomics data have been deposited in the ProteomeXchange Consortium via the PRIDE partner repository (https://www.ebi.ac.uk/pride/) with the data set identifier PXD060867. We freely provide the input files (initial coordinates, topologies, GROMACS mdp parameter file, and PLUMED inputs) to perform the MD simulations in this work, as well as the output simulation trajectories (in xtc format) that we generated. We supply a Jupyter notebook to reproduce all of our analyses, results, and the plots reported in this work. All the material is freely available in Zenodo with accession code 10.5281/zenodo.17205343. PLUMED input files are also available on the PLUMED-NEST under plumID:25.027, while the Jupyter notebook can also be straightforwardly consulted and downloaded at github.com/CompMedChemLab/project_saxs-xlms-md_rad. The entire output trajectory from the metad simulation, which together with the frame-by-frame weights determined via maxent represents the reweighted ensemble, can be found in the/metad/output subfolder in xtc format. The structures can also be directly accessed in PDB format in the/reweighted_ensemble_pdb folder, each labeled with the corresponding weight in the REMARK header line. The frame-by-frame weights associated with the reconstructed conformational ensemble can be found in a portable format in the/notebook/output_check subfolder; the weights can also be easily recomputed and saved using dedicated instructions in the notebook. In the notebook/output_check/out_cluster_analysis subfolder we also supply representative structures, i.e., cluster centroids, for the highest-weighted clusters from the reweighted ensemble in the form of individual PDBs, labeled with the corresponding cluster weight in the REMARK header line of each PDB; instructions to identify and save these structure files can also be found in the notebook. All MD simulations were performed with GROMACS 2023.1, patched with plumed 2.9. The VMD software version 1.9.4 was used for visualization, and analyses were conducted with MDtraj version 1.9.9, MDAnalysis Version 2.6.1, scikit-learn version 1.3.1.

## Notes

AlphaFold2 modeling of the RAD51–BRC4 complex; SLS binding tests; cross-linking trials and sample mass spectra of cross-linked peptides; single-structure models obtained via the SB-hySAXS scheme and respective computed SAXS spectra; timeseries of *R*_g_, RMSD and Lys–Lys distance during the Metadynamics simulations; residuals between computed and experimental SAXS spectra; summary of clusters with the highest weights, and corresponding distributions of the XL-MS distances; and full residue-wise contact matrix from the reweighted ensemble (PDF)
