# Supplementary material for: Dissecting the RAD51–BRC4 Interaction Landscape through Integrative Molecular Simulations and Experimental Biophysics
Source: J Chem Inf Model. 2025 Oct 22;65(21):11965–78. doi: 10.1021/acs.jcim.5c01639 (PMC12606624; doi:10.1021/acs.jcim.5c01639)
Supplement: Supplementary file 1 [file ci5c01639_si_001.pdf]

Supporting Information to

Dissecting the RAD51-BRC4 Interaction Landscape through Integrative Molecular Simulations and Experimental Biophysics

Authors

Veronica Bresciani[1,2,+], Francesco Rinaldi[1,+], Pedro Franco[3,4], Stefania Girotto[6], Andrea Cavalli[1,2,5], Julian D. Langer[3,4], Matteo Masetti[2,*], Mattia Bernetti[1,2,7,*]

[1] Computational and Chemical Biology, Istituto Italiano di Tecnologia, 16163, Genova, Italy

[2] Department of Pharmacy and Biotechnology (FABIT), Alma Mater Studiorum – Università di Bologna, 40126, Bologna, Italy

[3] Max Planck Institute of Biophysics, Max-von-Laue-Strasse 3, 60438 Frankfurt am Main, Germany

[4] Max Planck Institute for Brain Research, Max-von-Laue-Strasse 4, 60438 Frankfurt am Main, Germany

[5] Centre Européen de Calcul Atomique et Moléculaire (CECAM), Ecole Polytechnique Fédérale de Lausanne, 1015 Lausanne, Switzerland

[6] Structural Biophysics Facility, Istituto Italiano di Tecnologia, Genoa, 16163 Italy

[7] Department of Biomolecular Sciences (DISB), Università degli Studi di Urbino "Carlo Bo", Urbino, 61029, Italy

* Corresponding authors: matteo.masetti4@unibo.it, mattia.bernetti@uniurb.it

Supporting Figures

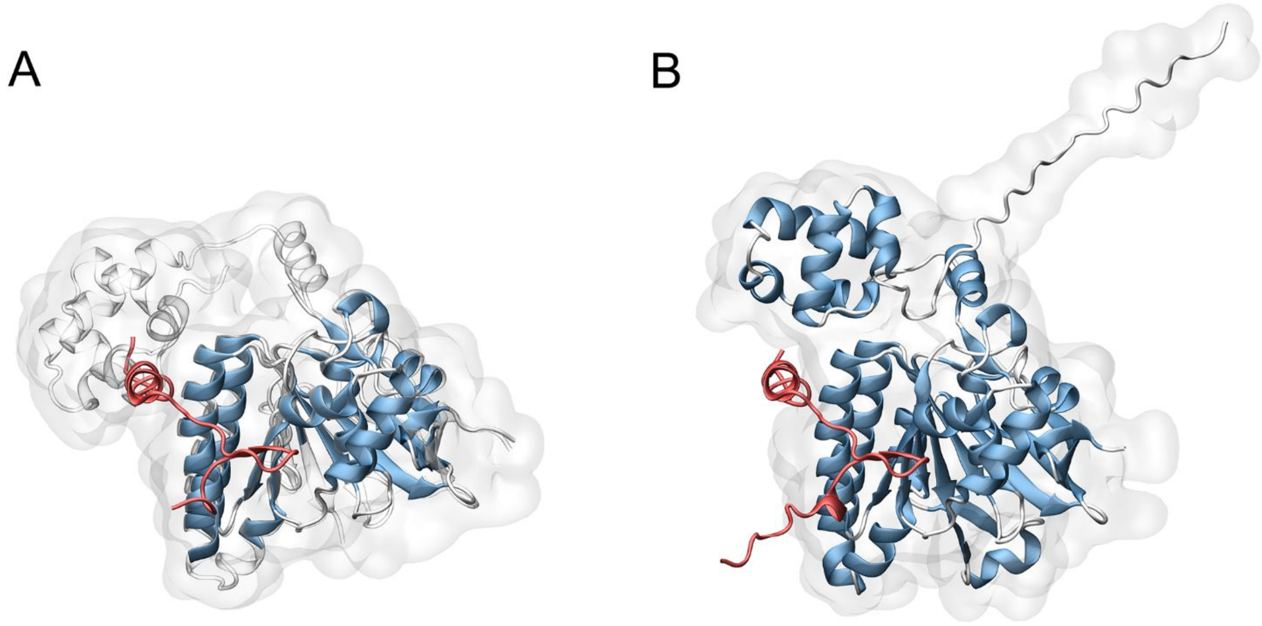

Figure S1. Structure of the RAD51-BRC4 complex. A) PDB entry 1N0W, featuring RAD51's C-ter (in blue) and BRC4 (in red), superposed with PDB entry 5NWL, featuring RAD51's C-ter and N-ter (in white) and lacking the BRC4 repeat. The superposition highlights how the N-ter domain and BRC4 are in overlap. B) The generated AlphaFold2 model, featuring RAD51's C-ter and N-ter (in blue) and the BRC4 repeat (in red). In the prediction, the N-ter is shifted to preserve BRC4 binding and avoid overlap with the repeat.

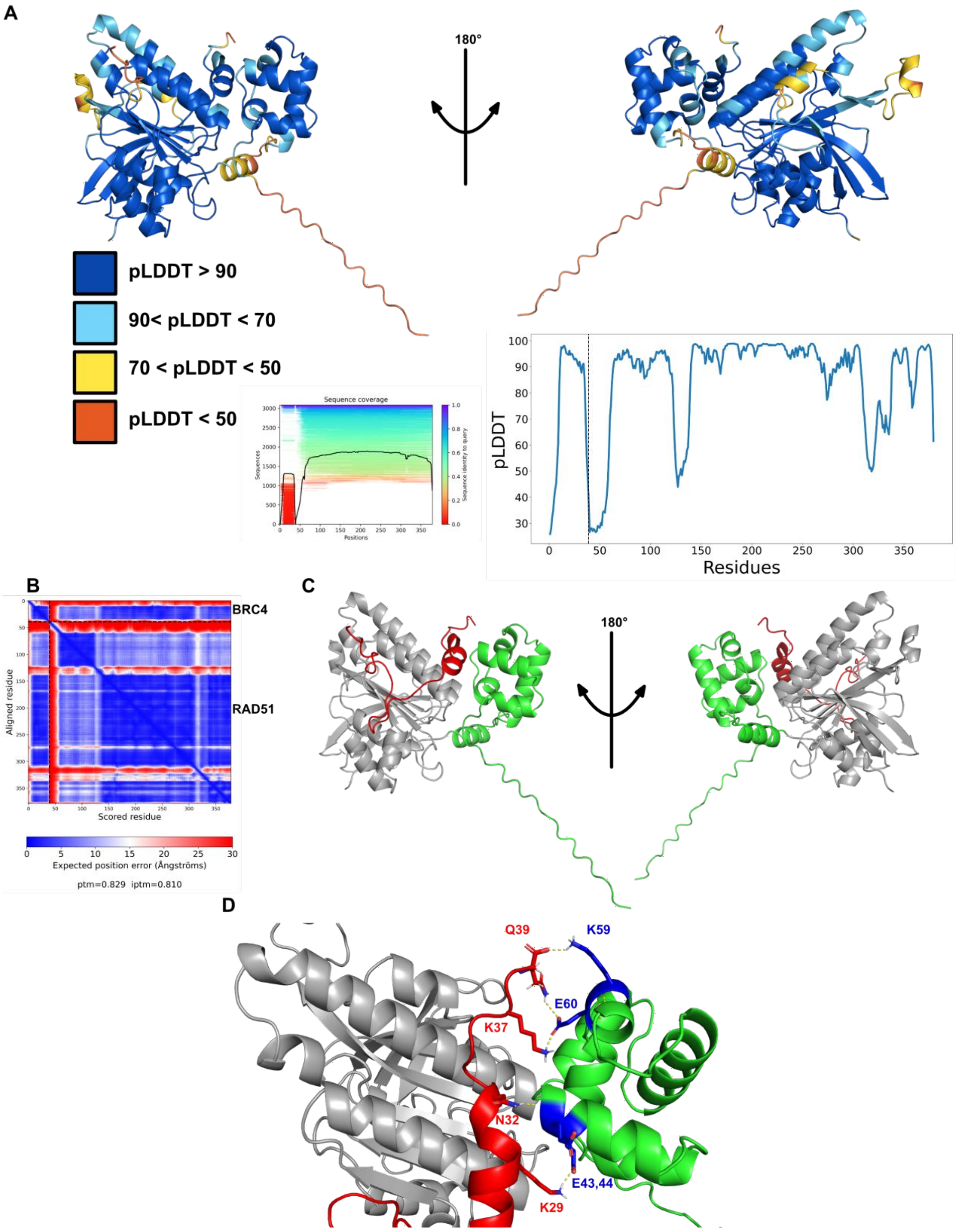

Figure S2. Alphafold 2.3 model of full length RAD51 monomer in complex with BRC4. A. Residues have been colored for local model confidence estimated with Predicted Local Distance Difference Test (pLDDT) representation (per-residue accuracy metric of the generated model) B. Predicted Align Error (PAE) plot, showing the expected positional error at residue x if the model is aligned on residue y. Right: Alphafold 2.3 Multiple Sequence Alignment. C. Residues have been colored by domain (green: N-terminal domain, grey: C-terminal domain) D. Analysis of polar interactions (hydrogen bonds) taking place between BRC4 and the RAD51 N-terminal domain predicted by Alphafold 2.3.

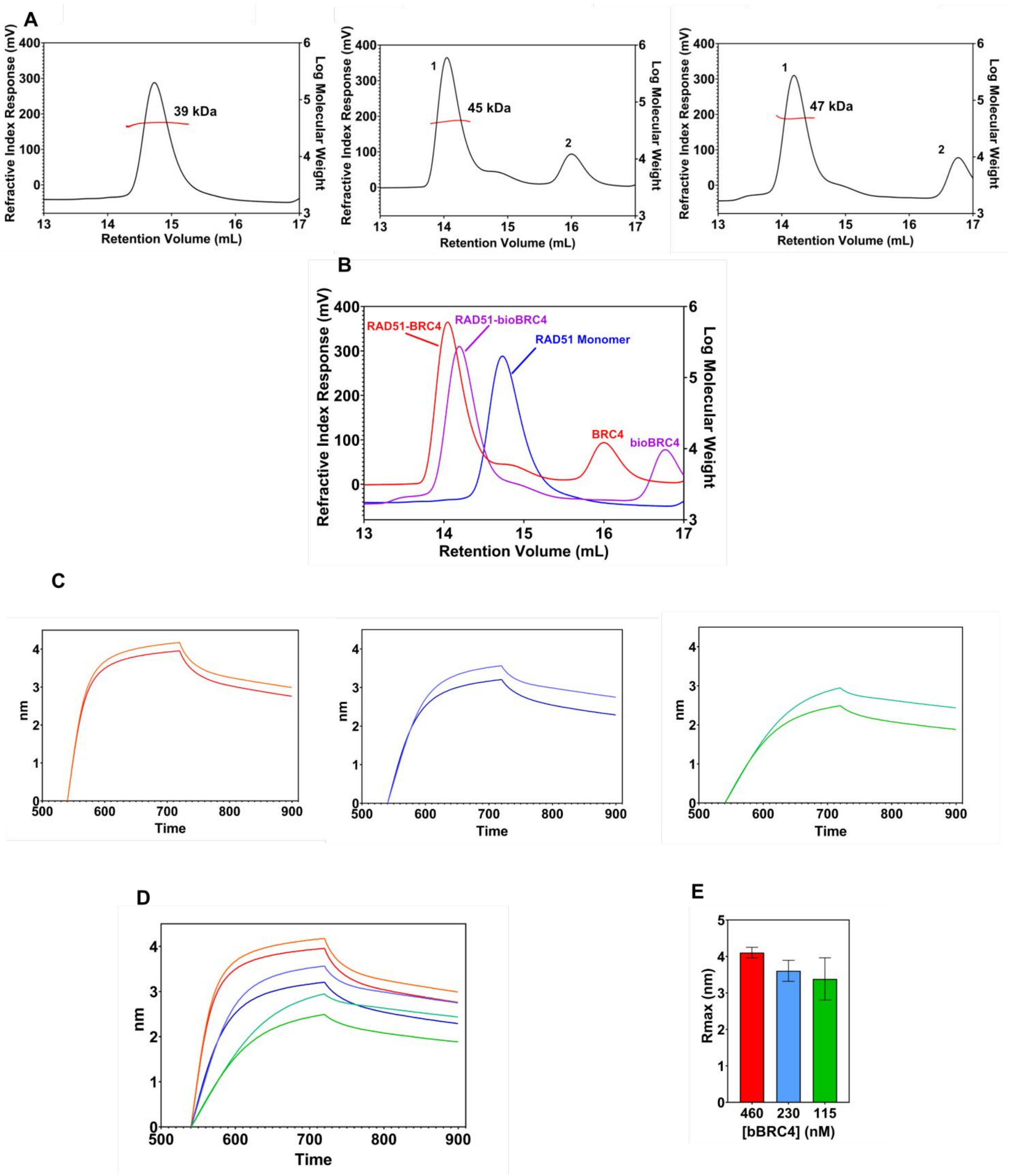

Figure S3. Binding tests of the biotinylated BRC4 peptide (bio-BRC4) to the monomeric His-RAD51 [F86E, A89E] A. SLS analysis in buffer containing 100 mM Na_2SO_4. Left: RAD51 [F86E, A89E] Middle: RAD51 [F86E, A89E] in presence of BRC4 peptide, peak 1 represents the RAD51-BRC4 complex, peak 2 represents the BRC4 peptide alone. Right: RAD51 [F86E, A89E] in presence of bioBRC4 peptide, peak 1 represents the RAD51-BRC4 complex, peak 2 represents the bioBRC4 peptide alone BRC4 peptide (green). B. Overlay

view of the samples in the absence (blue) or presence of BRC4 (red) and bioBRC4 peptide (purple). C. Two replicates of biolayer interferometry (BLI)sensorgrams showing the binding of monomeric His-RAD51[F86E, A89E] to bioBRC4 obtained at three different protein concentrations. Left: 460 nM, Middle: 230 nM, Left: 115 nM D. Overlay of all BLI sensorgrams E. Mean ± standard deviation of maximum response (R_{max}) obtained for BLI experiments carried out at the same protein concentration.

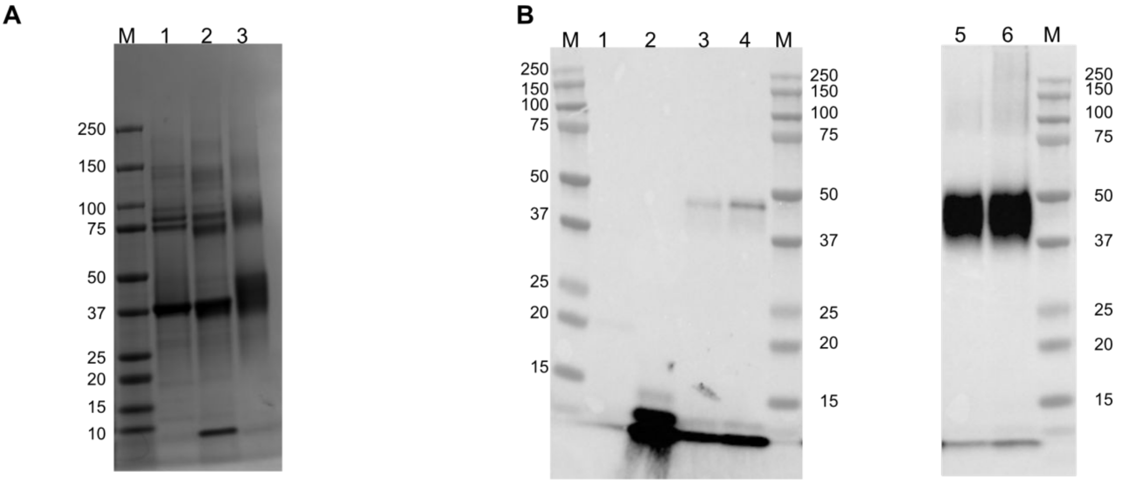

Figure S4. Monomeric RAD51 [F86E, A89E] bioBRC4 cross-linking trials. A. SDS Page gel Coomassie Blue Staining, M = Marker, 1 = RAD51 [F86E, A89E] 2 = RAD51:bioBRC4 complex 1:2 crosslinked with EDAC 0.2 % (w/v) 3 = RAD51:bioBRC4 complex 1:1 crosslinked with BS3 (1 mM) B. Western Blot detecting bio-BRC4 with Streptavidin-HRP M = Marker, 1 = RAD51 [F86E, A89E], 2 = bioBRC4, 3 = RAD51:bioBRC4 complex 1:1 4 = RAD51:bioBRC4 complex 1:2 5 = RAD51:bioBRC4 complex 1:1 BS3 1 mM 6 = RAD51:bioBRC4 complex 1:2 BS3 1 mM.

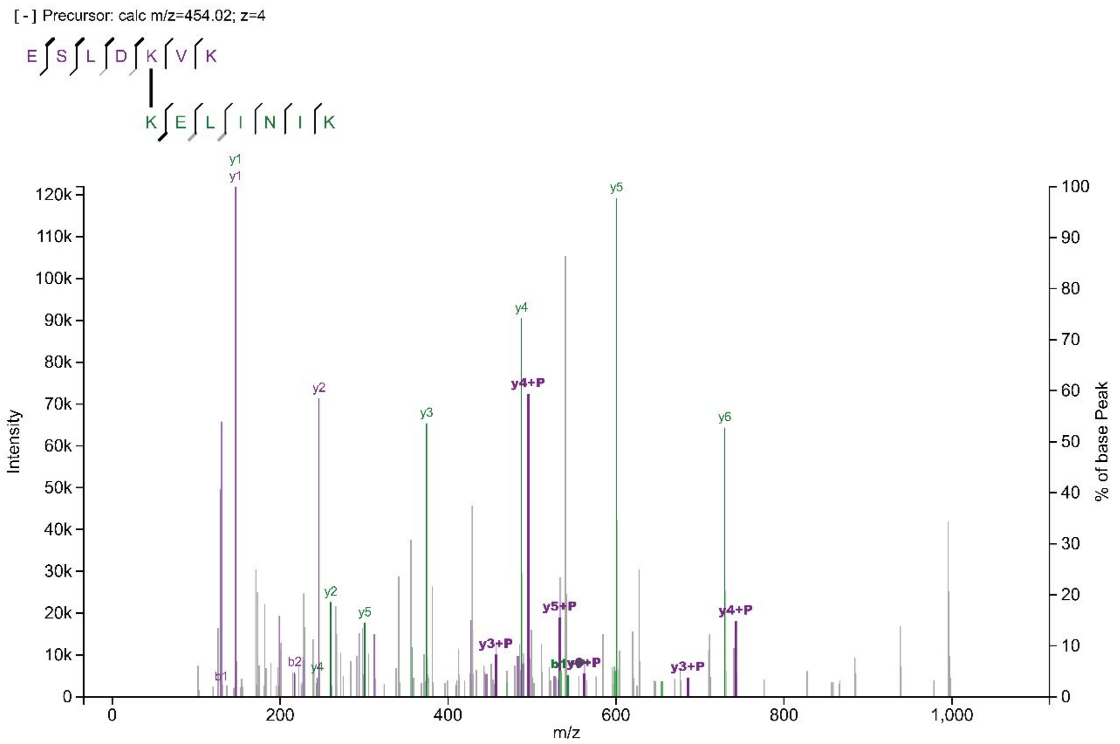

Figure S5. Example mass spectra of crosslinked peptides, evidencing the quality of the identifications.

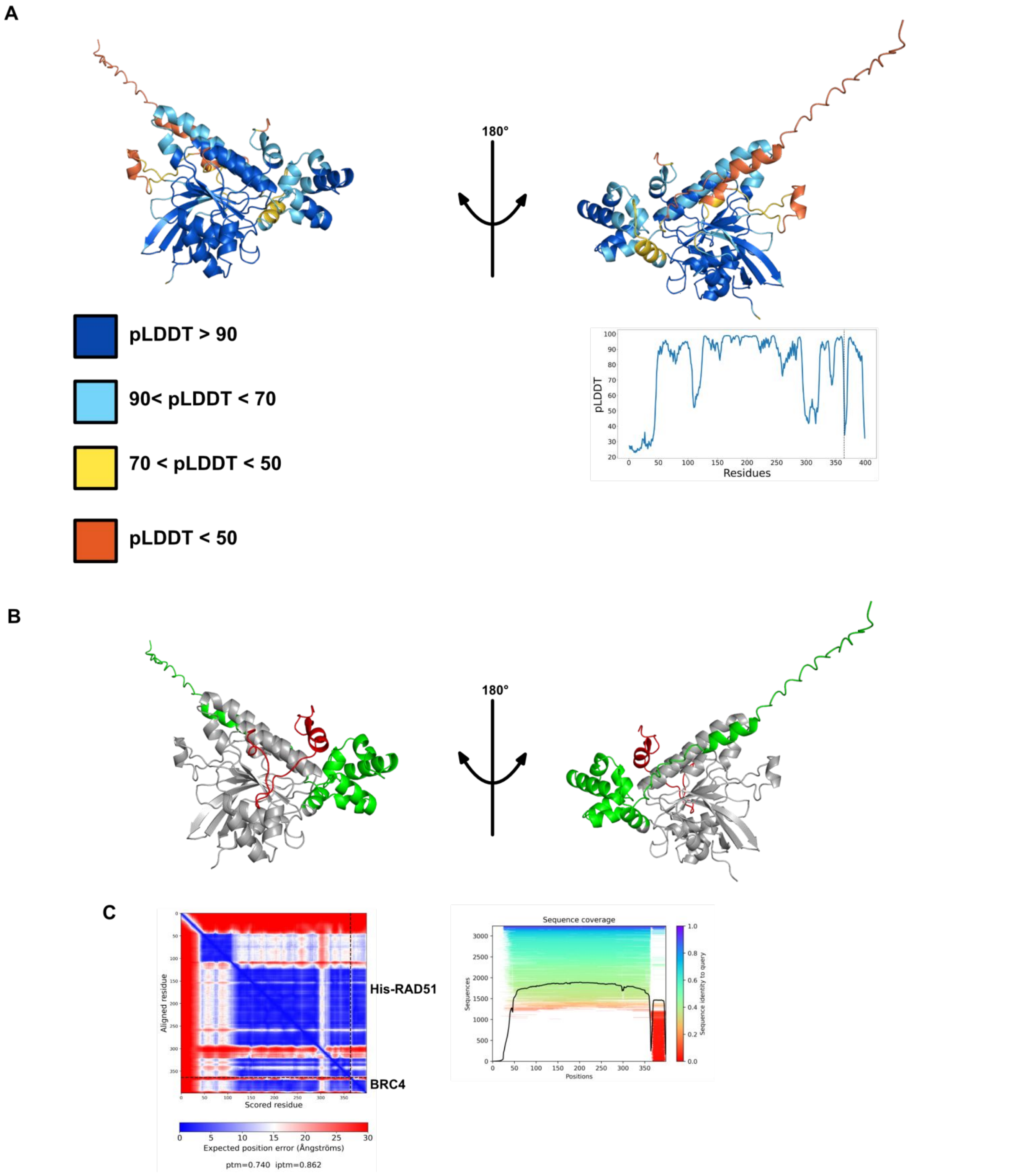

Figure S6. Alphafold 2.3 model of His-tagged full length RAD51 monomer in complex with BRC4. A. Residues have been colored for local model confidence estimated with Predicted Local Distance Difference Test (pLDDT) representation (per-residue accuracy metric of the generated model) B. Residues have been colored by domain (green: N-terminal domain, grey: C-terminal domain) C. Left: Predicted Align Error (PAE) plot, showing the expected positional error at residue x if the model is aligned on residue y. Right: Alphafold 2.3 Multiple Sequence Alignment.

A

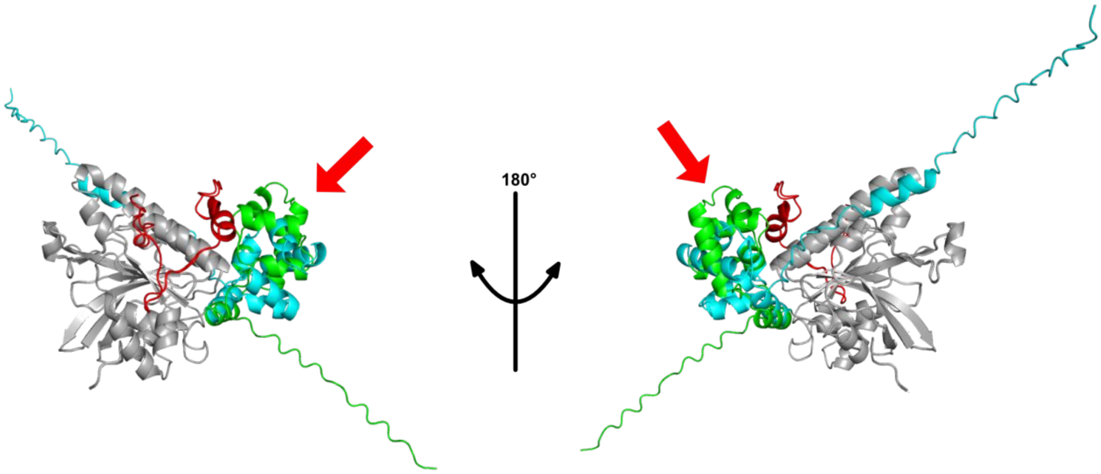

R.M.S.D. = 0.303

B

RAD51-BRC4

>A

GWHMKEPTLLGFHTASGKKVKIAKESLDKVKNLFDEKEQ

>B

GMAMQMQLEANADTSVEEESFGPQPISRLEQCGINANDVKKLEEAGFHTVEAVAYAPKKELINIKGISEAKADKIL
AEAAKLVPMGETTETEFHQRRSEIIQITTGSKELDKLLQGGIETGSITEMFGEFRTGKTQICHTLAVTCQLPIDRGG
GEGKAMYIDTEGTFRPERLLAVAERYGLSGSDVLDNVAYARAFNTDHQTQLLYQASAMMVESRYALLIVDSATAL
YRTDYSGRGELSARQMHLARFLRMLLRLADEFGVAVVITNQVVAQVDGAAMFAADPKKPIGGNIIAHASTTRLYL
RKGRGETRICKIYDSPCLPEAEAMFAINADGVGDAKD

HisRAD51-BRC4

>A

KEPTLLGFHTASGKKVKIAKESLDKVKNLFDEKEQ

>B

MGSSHHHHHHSSGLVPRGSHMLEDPMAMQMQLEANADTSVEEESFGPQPISRLEQCGINANDVKKLEEAGFH
TVEAVAYAPKKELINIKGISEAKADKILAEAAKLVPMGETTETEFHQRRSEIIQITTGSKELDKLLQGGIETGSITEMF
GEFRTGKTQICHTLAVTCQLPIDRGGGEGKAMYIDTEGTFRPERLLAVAERYGLSGSDVLDNVAYARAFNTDHQT
QLLYQASAMMVESRYALLIVDSATALYRTDYSGRGELSARQMHLARFLRMLLRLADEFGVAVVITNQVVAQVDGA
AMFAADPKKPIGGNIIAHASTTRLYLRKGRGETRICKIYDSPCLPEAEAMFAINADGVGDAKD

Figure S7. Overlay of the Alphafold 2.3 generated models. Overlay of the RAD51-BRC4 and of the His-RAD51-BRC4 complexes. Red arrows highlight the different arrangements of the RAD51 N-terminal domain in the two predicted models. In multiple colours different domains of RAD51 (green (RAD51-BRC4) and cyan (His-RAD51-BRC4) represent the RAD51 N-terminal domain while the C-terminal is displayed in grey) in the generated AF2 model and the BRC4 peptide (in red). The root mean square

deviation (R.M.S.D.) of the two models is displayed in the figure. B. Amino acidic sequence of the generated AF2 models.

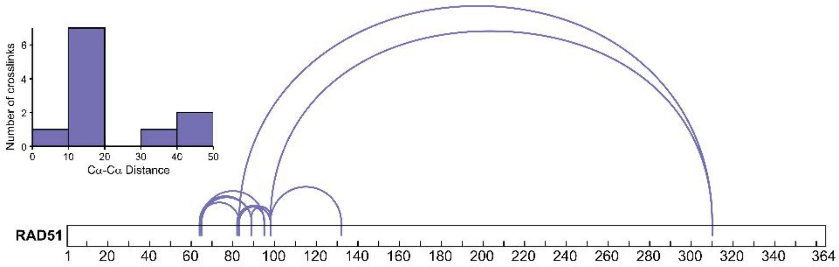

	Cα-Cα distance	
Crosslink	-His Tag	+His Tag
K64 - K83	18.24	18.33
K64 - K89	11.31	11.30
K65 - K89	7.59	7.61
K65 - K95	11.57	11.69
K82 - K98	10.60	10.44
K83 - K310	39.16	46.75
K83 - K95	11.89	11.80
K83 - K98	13.63	13.45
K89 - K98	15.91	15.96
K98 - K132	47.96	41.67
K98 - K310	43.96	48.69

Figure S8. RAD51 intramolecular cross-links identified in XL-MS analysis.

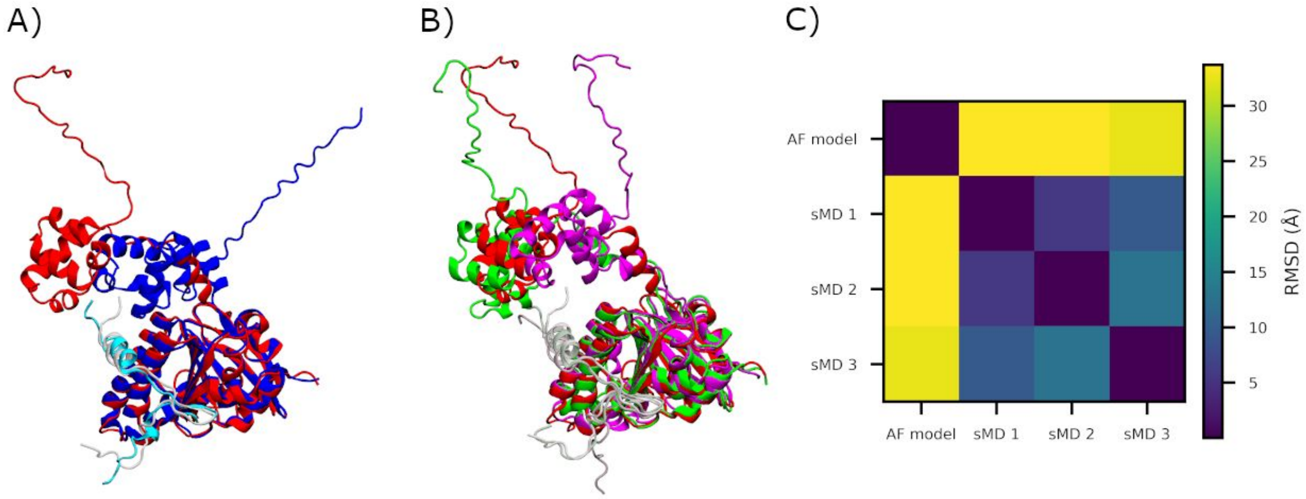

Figure S9. A) One model from the single structure model from replicate 1 of the steered MD simulations via the SB-hySAXS scheme (red) superposed to the AlphaFold model (blue), and B) single structure models obtained at the end of the three replicates of steered MD (red, green, and purple for replicates 1, 2, and 3, respectively). All structures are aligned on the heavy atoms of RAD51's C-ter domain. C) RMSD matrix between the three single structure models obtained at the end of the steered MD replicates and the AlphaFold model; the RMSDs were computed on the system's α carbons after optimal alignment on the α carbons of the C-ter domain.

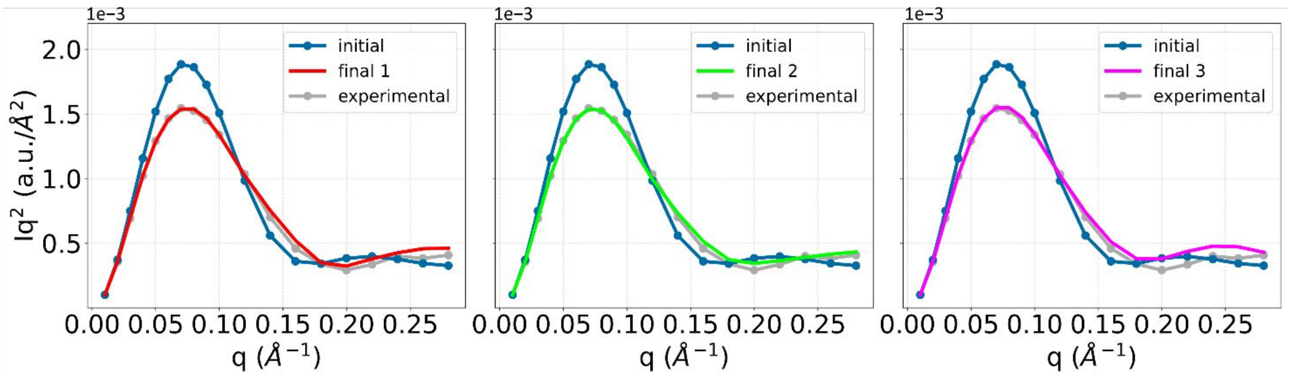

Figure S10. SAXS spectra in the Kratky form for the three replicates of steered MD using the SB-hySAXS scheme. The three panels report the spectrum computed at the end of the simulation (labelled final, and color coded according to figure S3B), compared with the starting structure used for the simulations, i.e. the AlphaFold model (in blue), and the experimental SAXS spectrum (in grey).

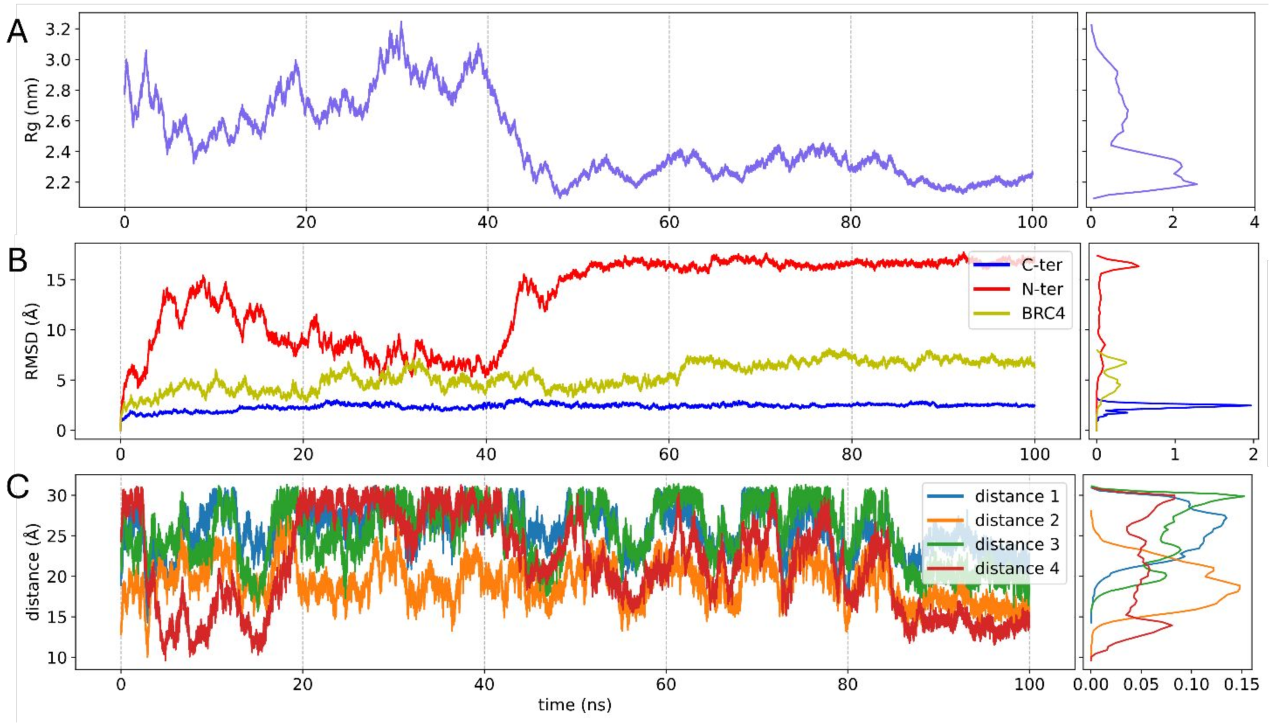

Figure S11. Timeseries and corresponding distributions along the metad simulation for A) the radius of gyration of the system, B) RMSDs of RAD51's N-ter and C-ter, and the BRC4 repeat, and C) distances between lysine pairs from the XL-MS experiments. RMSDs were computed on heavy atoms, after aligning separately on each of the three characters (N-ter, C-ter, and BRC4).

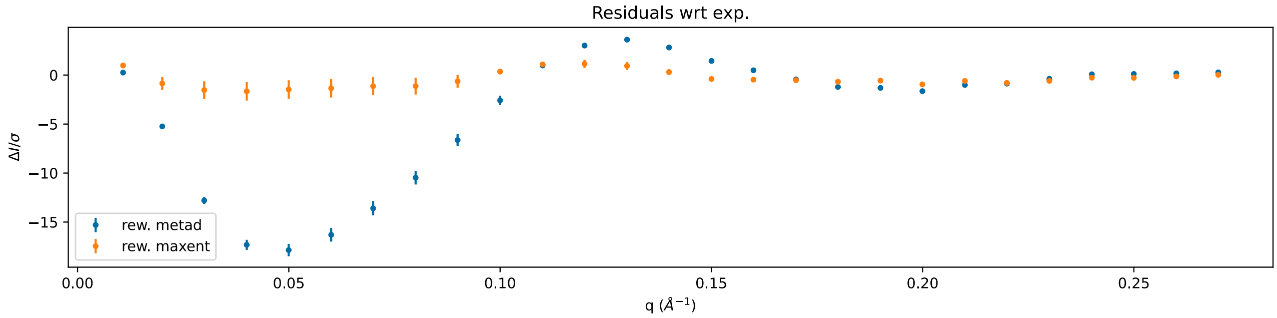

Figure S12. Residual between computed (metad and maxent-reweighted ensembles, in blue and orange, respectively) and experimental SAXS spectra.

Table S1. Results of the cluster analysis on the PCs using the QT algorithm, performed separately on compact and extended structures. Cluster indices, weights, and the weighted average of Rg are reported for the first 15 clusters with the highest weights.

	Cluster ID	weight	Rg (Å)
Compact (Rg < 24.7 Å)	1	0.01996	23.5
	2	0.01834	23.3
	3	0.01510	23.4
	4	0.01508	23.6
	5	0.01104	23.2
	6	0.01053	23.3
	7	0.00994	23.6
	8	0.00981	23.2
	9	0.00965	23.4
	10	0.00942	23.2
	11	0.00936	23.2
	12	0.00899	23.5
	13	0.00816	23.3
	14	0.00782	23.6
	15	0.00754	23.4
Extended (Rg > 24.7 Å)	1	0.01605	29.3
	2	0.01169	29.2
	3	0.01148	29.3
	4	0.00999	26.8
	5	0.00975	27.1
	6	0.00946	29.3
	7	0.00870	27.8
	8	0.00861	28.7
	9	0.00852	29.3
	10	0.00797	29.2
	11	0.00758	27.3
	12	0.00749	29.3
	13	0.00721	27.1
	14	0.00720	27.8
	15	0.00715	28.8

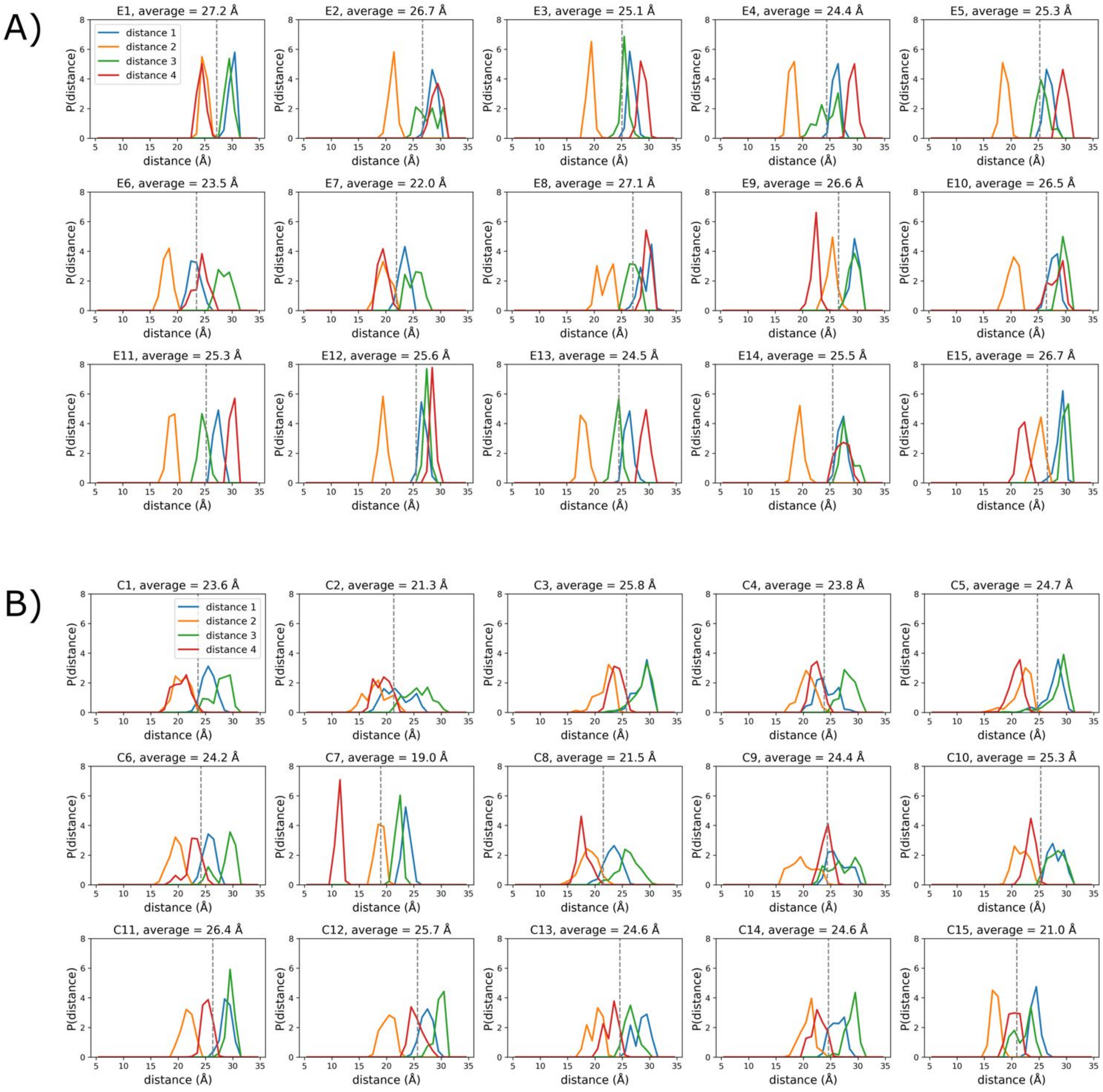

Figure S13. Distribution of the XL-MS distances in the main structural clusters of the reweighted ensemble. The four distance are colored consistently with Figure S11C, while their weighted average is shown as a vertical dashed grey line and is also reported in the title of each panel along with the cluster ID. The results for the first 15 clusters with the highest weights are shown for both A) extended and B) compact conformations.

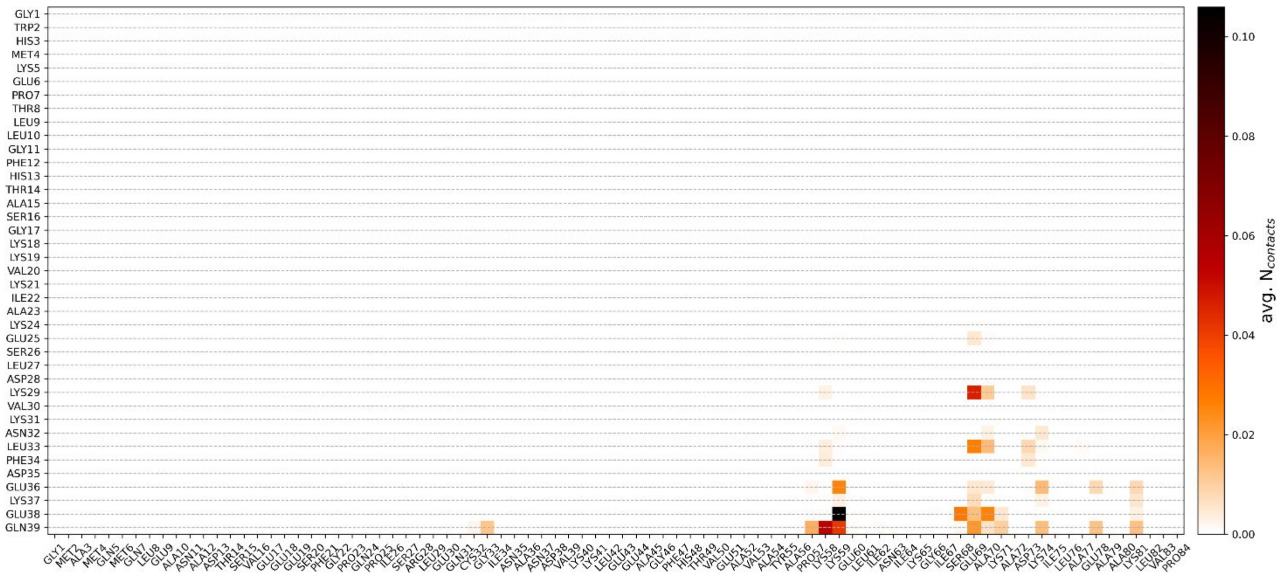

Figure S14. Residue-wise contact matrix between RAD51's N-ter and BRC4.
